# Cyclophilin A causes severe fever with thrombocytopenia syndrome virus-induced cytokine storm by regulating mitogen-activated protein kinase pathway

**DOI:** 10.3389/fmicb.2022.1046176

**Published:** 2022-12-01

**Authors:** Huaying Huang, Ke Jin, Ke Ouyang, Zhengyi Jiang, Zhan Yang, Nannan Hu, Yan Dai, Yaqin Zhang, Qian Zhang, Ying Han, Jie Zhao, Hong Lin, Chunhui Wang, Chunyan Wang, Xuewei Sun, Dafeng Lu, Jin Zhu, Jun Li

**Affiliations:** ^1^Department of Infectious Diseases, The First Affiliated Hospital of Nanjing Medical University, Nanjing, China; ^2^Department of Respiratory Diseases, Affiliated People's Hospital of Jiangsu University, Zhenjiang, China; ^3^Huadong Medical Institute of Biotechniques, Nanjing, China; ^4^Jiangsu Province Blood Center, Nanjing, China; ^5^Basic Medical College, Binzhou Medical University, Yantai, China; ^6^School of Public Health, Nanjing Medical University, Nanjing, China

**Keywords:** severe fever with thrombocytopenia syndrome, Cyclophilin A, Cyclosporine A, mitogen-activated protein kinase, infection

## Abstract

**Introduction:**

Severe fever with thrombocytopenia syndrome (SFTS) has become a global threat to public health since its first report in China in 2009. However, the pathogenesis of SFTS virus (SFTSV) in humans remains unclear. Also, there are no effective therapeutics for SFTS. Cyclophilin A (CyPA) regulates protein folding and trafficking involved in various viral infectious diseases, but its role in SFTSV infection has not been elucidated.

**Methods:**

We detected plasma CyPA levels in 29 healthy subjects and 30 SFTS patients by ELISA. In THP-1 cells and normal human peripheral blood mononuclear cells (PBMCs), SFTSV-induced extracellular CyPA (eCyPA) was also detected by ELISA. In THP-1, the effects of CyPA on Mitogen-activated protein kinase (MAPK) pathway and NF-κB were determined by Western blot. We validated the interaction between CypA and CD147 by human recombinant CyPA (hrCyPA) and the CD147 inhibitor. Effects of CyPA inhibitor Cyclosporine A (CsA) on cytokines and SFTSV replication in THP-1 cells was also detected. 8-week-old Interferon-α/β Receptor (IFNAR) knockout (IFNAR-/-) C57BL/6 mice were divided into mock group, 10^6^TCID_50_ SFTSV (Untreated) group and 10^6^TCID_50_ SFTSV+CsA (CsA-treated) group. The changes of body weight, animal behavior and survival time of each group were recorded. Blood samples were collected from tail vein regularly. After death, the liver, spleen, lung, kidney and brain were collected for pathological HE staining and SFTSV-NP immunohistochemical staining.

**Results:**

Compared to healthy subjects and SFTS patients in the febrile phase of the disease, plasma CyPA levels in SFTS patients at the multi-organ dysfunction (MOD) phase showed significantly elevated (*P* < 0.01). Extracellular CyPA activates the MAPK pathway by binding to CD147 in THP-1 infected with SFTSV. CsA inhibits the pro-inflammatory and promoting replication effects of CyPA after SFTSV infection in vitro. In vivo, CsA can prolong the survival time and delay the weight loss of SFTSV mice. CsA reduces multi-organ dysfunction in IFNAR−/− mice infected with SFTSV.

**Discussion:**

Our results indicate that CyPA is associated with SFTSV-induced cytokine storm, which can be a potential target for SFTS therapy.

## Introduction

Severe fever with thrombocytopenia syndrome (SFTS) is an emerging acute infectious disease first reported in China in 2009. Subsequent cases were also reported in South Korea, Japan, and Vietnam, with high mortality rates ranging from 6 to 30% (Shimojima et al., 2013; Kim et al., 2017; Tran et al., 2019; Liu and Gao, 2020). SFTSV poses a great public health threat globally due to its worldwide spread, high mortality, and human infectivity of the virus. The pathogenesis of the SFTS virus in humans is poorly understood, and effective treatments and vaccines for the virus are not yet available.

Previous case series reports indicate increased levels of some cytokines and chemokines such as IL-6, IL-10, IP-10, IFN-γ, TNF-α, and MCP-1 during the acute phase of SFTS (Deng et al., 2012; Cui et al., 2014; Ding et al., 2014; Liu et al., 2017; Song et al., 2017). On the contrary, other studies report the elevation of other cytokines and chemokines such as IL-1β, IL-8, MIP-1α, and MIP-1β during the acute phase of disease in fatal cases and the convalescent phase of non-fatal cases (Sun et al., 2012; Kwon et al., 2018). There is an association between the levels of pro-inflammatory cytokines and chemokines and disease severity (Deng et al., 2012). In addition, studies have shown that high viral load at hospitalization is a predictor of poor outcomes in SFTS patients (Zhang et al., 2012; Li et al., 2014; Hwang et al., 2017; Kwon et al., 2018). Therefore, there is a critical need for therapies targeting the inhibition of SFTS cytokine storm and SFTSV replication.

There are no prospective randomized trials of treatment strategies that have proven to be effective against SFTS. Ribavirin is the only drug approved for the treatment of respiratory syncytial virus and is also often used to study the therapeutic effects of other viral infections. Clinical studies of ribavirin use for the treatment of SFTS have shown conflicting results (Liu et al., 2013a; Cui et al., 2014; Li et al., 2018). Thus, ribavirin is not currently considered as an effective treatment option. Favipiravir (T-705) is a pyrazine derivative and broad-spectrum antiviral drug developed in Japan. The efficacy and safety of Favipiravir in the treatment of SFTS have been previously demonstrated (Suemori et al., 2021). However, more evidence is needed for further verification. Therefore, there is an urgent need to explore new anti-SFTS therapies.

CyPA was first isolated from bovine thymocytes as a cytoplasmic protein that primarily binds to CsA (Handschumacher et al., 1984). During infection, oxidative stress, and hypoxia, CyPA is secreted extracellularly by mononuclear macrophages, vascular endothelial cells, and vascular smooth muscle cells (Sherry et al., 1992; Jin et al., 2000; Cao et al., 2019). eCyPA plays a regulatory role during inflammation in various diseases, such as periodontitis, infection, atherosclerosis, diabetes, and cancer proliferation and metastasis (Jin et al., 2000; Li et al., 2006; Ohtsuki et al., 2017; Wang et al., 2019; Cao et al., 2020; Koh et al., 2021). MAPK modulates signaling cascades and transmits extracellular signals to intracellular targets. Therefore, MAPK cascades are central signaling elements that regulate basic processes, including cell proliferation, differentiation, and stress responses (Keshet and Seger, 2010; Plotnikov et al., 2011). Activation of ERK1/2, NF-κB, JNK, and p38 MAPK pathways have been observed in different cell types after stimulation with CyPA (Yurchenko et al., 2002; Jin et al., 2004). In viral infections, CyPA promotes or inhibits viral replication. CyPA plays a vital role in the propagation of viruses such as Hepatitis B virus (HBV), Hepatitis C virus (HCV), Vaccinia virus (VV), Human immunodeficiency virus type 1 (HIV-1), and coronavirus (CoV; Dawar et al., 2017). Conversely, CyPA can inhibit the replication of influenza virus and rotavirus (RV) by interacting with viral proteins (He et al., 2013; Liu et al., 2013b).

The role of CyPA in the regulation of inflammation and viral replication after SFTSV infection has not been reported. In this study, we observed high detection of CyPA in the plasma of SFTS patients. The pro-inflammatory and pro-replicative effects of CyPA in SFTSV-infected THP-1 cells and the inhibitory effect of CsA were investigated. In addition, we explored the efficacy of CsA in the treatment of SFTSV infection using a lethal mouse model of SFTS.

## Results

### Expression pattern of CyPA in the plasma of SFTS patients

**Figure 1:**
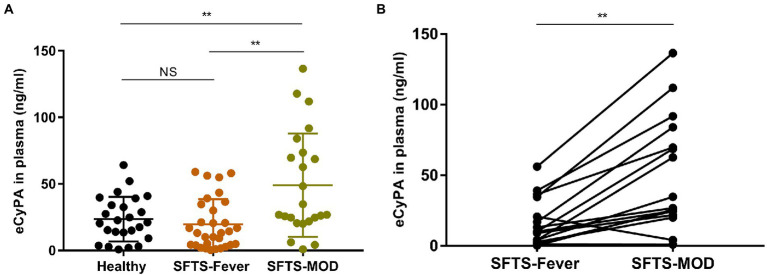
Expression pattern of Cyclophilin A (CyPA) in the plasma of SFTS patients. **(A)** Plasma CyPA detection levels as measured by ELISA in 29 healthy subjects and 30 SFTS patients. **(B)** Paired analysis of changes in the expression of CyPA in the plasma of 16 SFTS patients between the febrile and MOD phases. ^**^p < 0.01.

Analysis of plasma CyPA detection levels in 29 healthy subjects and 30 SFTS patients showed significantly elevated plasma CyPA levels in SFTS patients at the multi-organ dysfunction (MOD) phase compared to healthy subjects and SFTS patients in the febrile phase of the disease (Figure 1A). Paired analysis of SFTS patients in the febrile and MOD stages showed an increasing trend of plasma CyPA levels during the MOD phase (Figure 1B).

### Cyclosporine A inhibits the pro-inflammatory effect of CyPA after SFTSV infection *in vitro*

**Figure 2:**
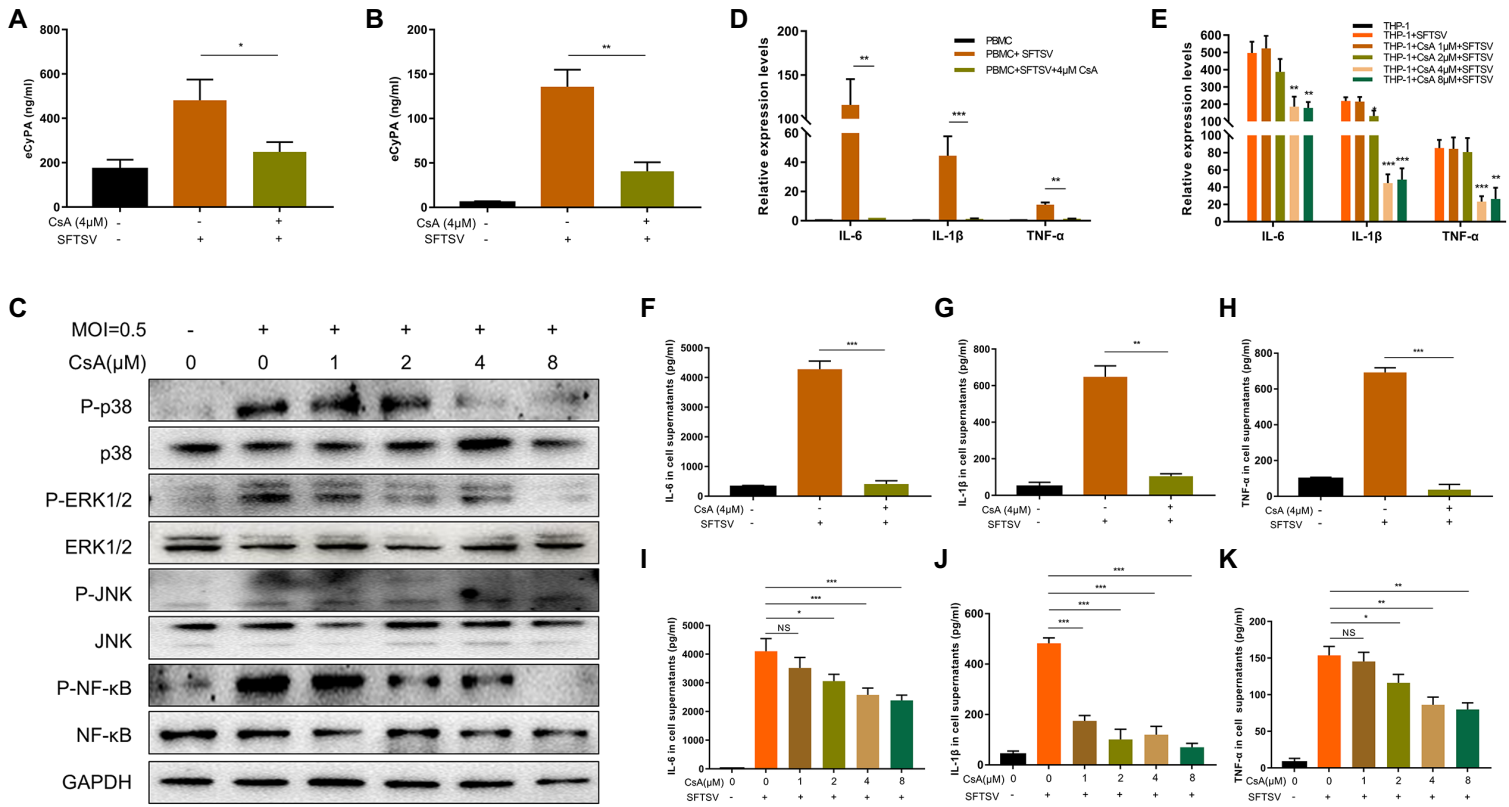
CsA inhibits the pro-inflammatory effects of CyPA after severe SFTSV infection. Normal human PBMCs **(A)** and THP-1 cells **(B)** were divided into blank group, SFTSV-infected (MOI = 0.5) 12 h group, 4 μM CsA pretreatment for 12 h, and SFTSV-infected 12 h (MOI = 0.5) group. The expression of CyPA in the supernatant of cells in each group was measured by ELISA. **(C)** THP-1 cells were divided into blank group, SFTSV-infected (MOI = 0.5) 12 h group, CsA pretreatment at different concentrations for 12 h, and SFTSV-infected 12 h (MOI = 0.5) group. The total protein in each group of cells was extracted to detect MAPK pathway activation by Western blot. The mRNA levels of IL-6, IL-1β, and TNF-α were measured by qRT-PCR in PBMCs **(D)** and THP-1 **(E)**. The protein levels of IL-6, IL-1β, and TNF-α in the supernatants of PBMCS **(F–H)** and THP-1 **(I–K)** were correspondingly collected and measured by ELISA. **(A,B,D–K)**. The values shown represent the mean ± standard error of three independent experiments. ^*^p < 0.05; ^**^p < 0.01; ^***^p < 0.001. **(C)** One representative experiment is shown.

Studies have shown that PBMCs are the target cells of SFTSV infection (Qu et al., 2012; Peng et al., 2016). Therefore, we selected THP-1 cells and normal human PBMCs for use in our experiments. The toxicity of CsA on THP-1 cells was first investigated using a cell proliferation kit (CCK8) and the results showed no difference in the toxicities of CsA between 0 μM and 8μΜ concentrations (Supplementary Figure S1). To investigate the expression of eCyPA in normal human PBMCs and THP-1 cells after an SFTSV infection, CyPA levels in cell supernatants were quantified by ELISA after SFTSV infection (multiplicity of infection [MOI] = 0.5) for 12 h. The expression levels of eCyPA in both PBMCs (Figure 2A) and THP-1 cells (Figure 2B) were significantly increased after SFTSV infection. However, we noticed that the expression levels of eCyPA in both PBMCs (Figure 2A) and THP-1cells (Figure 2B) were significantly reduced when cells pretreated with CsA at 4 μM for 12 h were infected with SFTSV (MOI = 0.5) for another 12 h.

Studies have shown that eCyPA modulates disease development by regulating the MAPK pathway (Li et al., 2006; Xiao et al., 2016; Guo et al., 2018; Li et al., 2020). To determine whether extracellular CyPA could activate the intracellular MAPK pathway after SFTSV infection, we divided THP-1 cells into three different groups: blank group, SFTSV-infected 12 h group, and SFTSV-infected 12 h group after pretreatment with different concentrations of CsA. We observed differential regulation of the phosphorylation levels of P38, ERK1/2, JNK, and NF-κB after SFTSV infection, while CsA pretreatment inhibited MAPK pathway phosphorylation (Figure 2C). The corresponding gray value analysis is shown in Supplementary Figure S2A.

We further quantified the expression levels of inflammatory cytokines in intracellular and cell supernatants of the different subgroups. The results showed a significant increase in both mRNA and protein levels of IL-6, IL-1β, and TNF-α in PBMCs (Figures 2D, F–H) and THP-1 cells (Figures 2E, I–K) after SFTSV infection. Conversely, the mRNA and protein expression levels of these inflammatory cytokines were significantly decreased after CsA pretreatment.

### Extracellular CyPA activates the MAPK pathway by binding to CD147 in THP-1 cells infected with SFTSV

**Figure 3:**
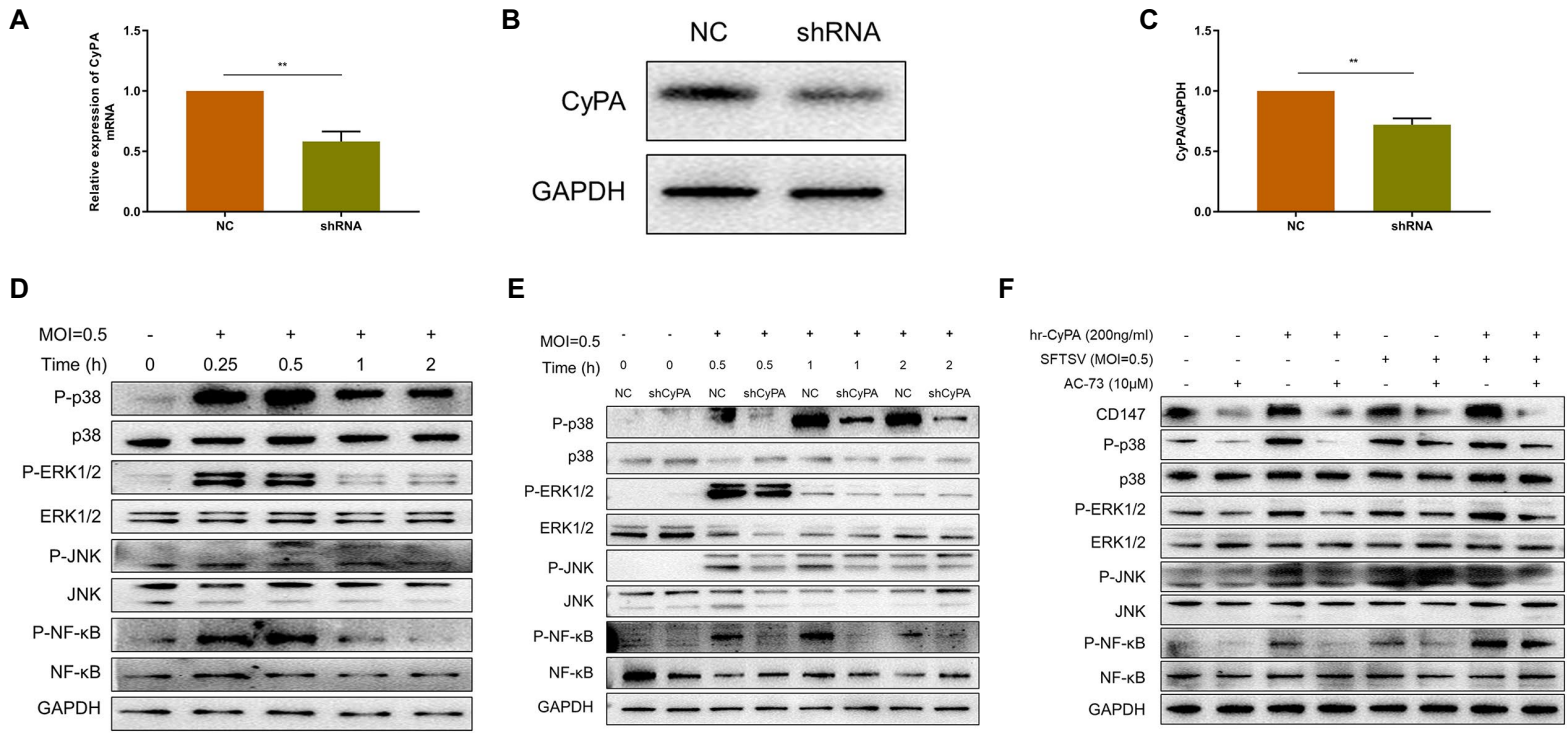
Extracellular CyPA binds to CD147 to activate the MAPK pathway in SFTSV-infected THP-1 cells. **(A)** The expression of CyPA mRNA as detected by qRT-PCR in THP-1 cells transfected with the sh-CyPA, compared with NC cells. **(B)** The CyPA protein level as detected by Western blot in sh-CyPA compared with NC cells. **(C)** The gray value analysis of **(B)**. **(D)** Western blot analysis of the indicated proteins in SFTSV-infected THP-1 lysates. **(E)** WB analysis of the indicated proteins in SFTSV-infected sh-CyPA and NC cell lysates at different times. **(F)** THP-1 cells were divided into blank group, AC-73 treated 4 h group, SFTSV or hrCyPA treated 0.5 h group, and co-treatment groups. Total cell lysates of each group were extracted for WB. **(A,C)** The values shown indicate the mean ± standard error of three independent experiments. ^**^p < 0.01. **(D–F)** One representative experiment is shown.

To confirm the role of CyPA in SFTSV infection, we knocked down the expression of CyPA in THP-1 cells using the short hairpin RNA (shRNA) technique. The knockdown efficiency was verified by qRT-PCR (Figure 3A) and WB (Figures 3B,C). Infection of THP-1 cells with SFTSV (MOI = 0.5) at different time points showed that the increase in MAPK pathway phosphorylation was most evident after 0.5 h of infection (Figure 3D). The corresponding gray value analysis is shown in Supplementary Figure S2B.

CyPA knockdown (sh-CyPA) and negative control (NC) THP-1 cells were then infected with SFTSV at different times. We observed that compared with NC, the phosphorylation of the MAPK pathway in sh-CyPA was significantly inhibited after SFTSV infection (Figure 3E). The corresponding gray value analysis is shown in Supplementary Figures S2C–F.

Since there was a relationship between extracellular CyPA expression and activation of the MAPK pathway, we further investigated whether this correlation depended on the membrane receptor CD147 of CyPA. The toxicities of hrCyPA and CD147 inhibitor AC-73 on THP-1 cells were studied using CCK8. The results demonstrated no differences between the toxicities of hrCyPA at 0 and 200 ng/ml and between the toxicities of AC-73 at 0 and 10 μM (Supplementary Figure S1). The MAPK pathway was activated when THP-1 cells were treated with hrCyPA or infected with SFTSV for 0.5 h; however, pretreatment with AC-73 for 4 h significantly inhibited MAPK pathway activation (Figure 3F).

### Cyclophilin A promotes SFTSV replication in THP-1 cells

**Figure 4:**
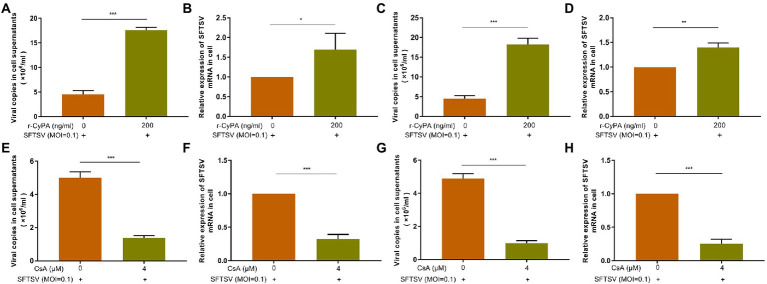
CyPA promotes the replication of SFTSV in THP-1 cells. In the experimental group, THP-1 cells were pretreated with hr-CyPA for 2 h and then infected with SFTSV for 48 h, after which the expression of SFTSV in the supernatant **(A)** and intracellular fluid **(B)** was determined. In the experimental group, THP-1 cells were infected with SFTSV for 2 h and then treated with hr-CyPA for 48 h, and the expression of SFTSV in the supernatant **(C)** and cells **(D)** was measured. In the experimental group, THP-1 cells were infected with SFTSV for 48 h after 2 h CsA pretreatment, and the expression of SFTSV in the supernatant **(E)** and intracellular fluid **(F)** was determined. In the experimental group, THP-1 cells were treated with CsA for 48 h after 2 h of SFTSV infection, and the expression of SFTSV in the supernatant **(G)** and cells **(H)** was measured. The values represent the mean ± standard error of three independent experiments. ^**^p < 0.01, ^***^p < 0.001.

The expression levels of SFTSV in THP-1 cell supernatant (Figures 4A,C) and intracellular (Figures 4B,D) were higher in the hrCyPA-treated group than in the hrCyPA-untreated group irrespective of whether hrCyPA was added 2 h before (Figures 4A,B) or 2 h after SFTSV infection (Figures 4C,D). On the contrary, the expression levels of SFTSV in THP-1 cell supernatants (Figures 4E,G) and intracellular (Figures 4F,H) were lower in the CsA-treated group than in CsA-untreated group, regardless of whether CsA was added 2 h before (Figures 4E,F) or 2 h after SFTSV infection (Figures 4G,H).

### Cyclosporine A effectively inhibits SFTSV infection *in vivo*

We have preliminarily demonstrated in *in vitro* experiments that: 1. eCyPA binds to CD147 to activate the MAPK pathway involved in SFTSV-induced cytokine secretion. 2. CyPA enhances SFTSV replication. 3. CsA inhibits the pro-inflammatory and viral replication effects of CyPA. We further explored whether inhibition of CyPA *in vivo* could also impede SFTSV-induced pro-inflammatory cytokine release and SFTSV viral replication.

We had previously constructed a lethal model of IFNAR^−/−^ mice using JS14 strain of SFTSV and successfully determined that 10 mg/kg/day of CsA has no significant toxic effect on IFNAR^−/−^ mice (Supplementary Figure S3). The untreated IFNAR^−/−^ mice were subcutaneously (s.c.) injected with 1.0 × 10^6^ 50% tissue culture infective doses (TCID_50_) of SFTSV on day 0. The CsA-treated mice were intraperitoneally (i.p.) injected with CsA at the dose of 10 mg/kg/day for five consecutive days starting 1 h post-infection.

**Figure 5:**
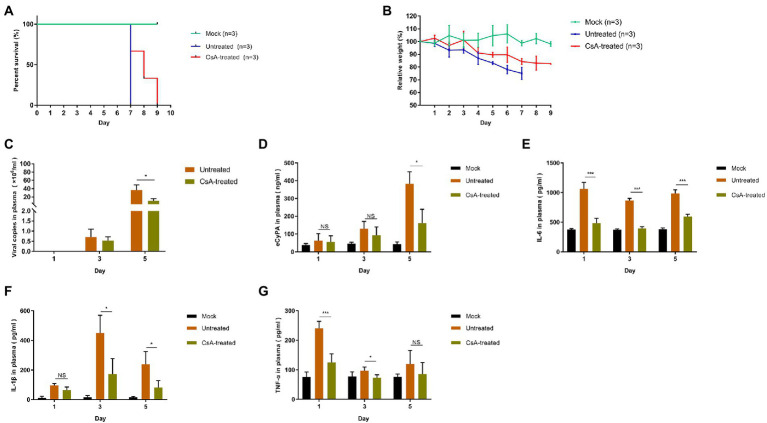
Treatment of SFTSV-infected IFNAR^−/−^ mice with CsA. **(A)** Mice in untreated and CsA-treated groups were inoculated s.c. with 1.0 × 10^6^ TCID_50_ of SFTSV, while the mock mice were inoculated s.c. with 100 μl DMEM. In the CsA-treated group, three mice were treated with 10 mg/kg/day CsA for five consecutive days. Survival curves were determined using GraphPad Prism7. **(B)** The relative weight is shown as means with standard deviations. **(C)** SFTSV RNA levels in plasma samples collected at 1,3, or 5 days post-infection as determined by qRT-PCR. **(D)** The levels of CyPA in plasma samples were detected by ELISA. **(E–G)** The protein levels of proinflammatory cytokines in plasma samples as detected by ELISA. Significance was determined relative to the untreated group: ^*^p < 0.05, ^**^p < 0.01, ^***^p < 0.001, NS, no significance.

The body weight of mice in the untreated group decreased significantly from the second day post-infection, and all mice died on day 7 after infection. The body weight of mice in CsA-treated group decreased from day 4 after infection and died on days 7, 8, and 9 after infection (Figures 5A,B). A quantitative RT-PCR assay was used to quantify the SFTSV viral copies in the blood of different mice. Compared with the untreated mice, there was a decline in the number of SFTSV virus copies in the plasma of CsA-treated mice on day 3 post-infection, and the degree of decrease on day 5 after infection was statistically significant (Figure 5C). In addition, we determined the expression of plasma CyPA in each group of mice (Mock, Untreated, and CsA-treated mice; Figure 5D). The expression levels of plasma CyPA in the CsA-treated group began to decline on day 3 post-infection, and the degree of reduction on day 5 after infection was significantly lower compared to the untreated group of mice (Figure 5D).

We also compared the expression levels of plasma inflammatory cytokines between the groups of mice. The plasma levels of IL-6, IL-1β, and TNF-α were higher in the untreated group than in the mock group on days 1, 3, and 5 post-infection. However, the levels of these plasma cytokines in the CsA-treated group were lower than in the untreated mice and the levels of decline varied with each cytokine (Figures 5E–G). IL-6 levels were the most significantly different between CsA-treated and untreated mice (Figures 5E–G).

### Cyclosporine A reduces multi-organ dysfunction in IFNAR^−/−^ mice infected with SFTSV

**Figure 6:**
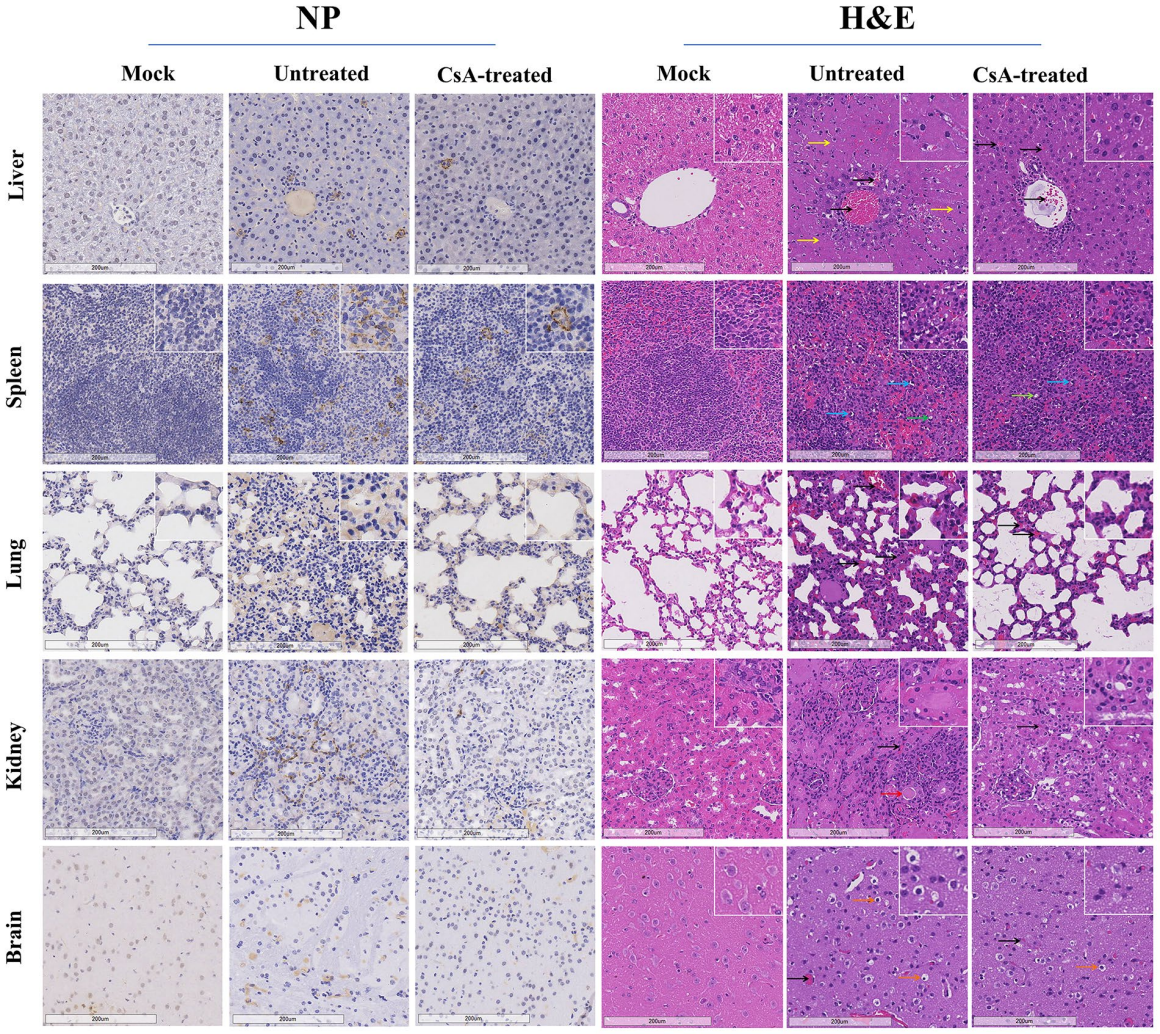
Pathology of IFNAR^−/−^ mice infected with SFTSV. Immunohistochemical (left) and histopathological (right) examinations of the tissues collected from the mock, untreated and CsA-treated mice. Tissues were collected from mice on day 7 (untreated mice) or day 9 (mock and CsA-treated mice). Bars, 200 μm. The black arrows indicate hyperemia, yellow arrows indicate necrosis, red arrows indicate protein cast, blue arrows indicate lymphocyte pyknosis, green arrows indicate lymphoid nucleus deletion, while orange arrows indicate neuronal edema.

Immunohistochemical analysis of liver, spleen, lung, kidney, and brain tissues showed significant upregulation of SFTSV nucleoprotein (NP) in untreated mice, while positive expression of SFTSV NP was only occasionally observed in the CsA-treated group (Figure 6 left). Histopathological analyses performed in each group of mice revealed differences between untreated and CsA-treated mice. For instance, analysis of the liver showed extensive necrosis and vascular stasis in the liver of untreated mice compared to the CsA-treated mice that exhibited only slight vascular stasis. Analysis of the white marrow portion of the spleens showed evidence of lymphocyte deficiency and sequestration in the untreated group, and the degree of these changes was remarkably reduced in the CsA-treated group. In the untreated group, the lung exhibited widening of alveolar septum and hyperemia of superior septal capillaries, the kidney showed interstitial congestion and partial renal tubular protein type, the brain showed neuronal edema and vascular congestion, while the above lesions were alleviated in the corresponding organs of CsA-treated group(Figure 6 right).

## Discussion

Cytokine storm and high viral load are the two major prognostic indicators of SFTS disease. Therefore, there is a need to explore the mechanisms for countering these syndromes. Previous studies have established that CyPA modulates inflammation in various diseases and also facilitates the propagation of viruses. However, the role of CyPA in the pathogenesis and viral replication of SFTSV has not been elucidated. Here, we found that plasma CyPA levels in SFTS patients were significantly correlated with disease severity. Thus, we hypothesized that CyPA is involved in cytokine storm induction following SFTSV infection. MAPK cascades are key signaling pathways that regulate diverse cellular processes, including proliferation, differentiation, apoptosis, and stress responses. Studies in different types of cells show that eCyPA activates MAPK pathway. In this study, it was verified that eCyPA interacts with CD147 in SFTSV-infected THP-1 cells to regulate MAPK pathway, which then upregulate the expression of IL-6, TNF-α, and IL-1β.

Many studies have shown that CyPA is a crucial facilitator of viral infection and that CyPA can embed viral proteins to enhance replication of various viruses (Liao et al., 2021). Here, we demonstrated that the expression of extracellular and intracellular SFTSV was significantly elevated by the addition of hrCyPA in THP-1 cells compared to controls, both before and after SFTSV infection. In contrast, the addition of the CyPA inhibitor CsA, before and after SFTSV infection, significantly decreased SFTSV copy numbers in culture supernatants and in THP-1 cells compared to the control group. These results indicate that CyPA can promote SFTSV replication; however, the specific mechanisms by which CyPA fosters SFTSV replication need to be explored further.

The current mouse models for studying the fatal SFTSV disease are IFNAR^−/−^ mice, newborn mice, and mitomycin-treated mice (Liu et al., 2014; Gowen et al., 2016; Xiao-Ping et al., 2020). Since the hematological manifestations of IFNAR^−/−^ mice, such as lymphopenia and thrombocytopenia, are similar to those of humans (Keita et al., 2017), these mice are an ideal animal model for studying the pathogenesis of SFTSV infection. In this study, we randomly grouped IFNAR^−/−^ C57BL/6 mice and then infected them with 10^6^TCID_50_ SFTSV based on previous results (Tani et al., 2016). Compared to the untreated group, the CsA-treated mice showed prolonged survival and delayed weight loss. Reduced organ inflammatory lesions and SFTSV NP expression indicate that CsA could attenuate SFTSV-induced cytokine storm and prevent viral replication *in vivo*. In conclusion, this is the first study to demonstrate the correlation between CyPA and SFTS cytokine storm *in vitro* and *in vivo*. Our findings suggest that CyPA is a potential new therapeutic target for SFTS treatment.

However, this study has the following shortcomings: 1. The sample size of SFTS patients was small, thus should be expanded to supplement the data. 2. IL-6 and TNF-α were significantly elevated on day one after infection, and the expression trends of plasma cytokines in mice on days 1, 3, and 5 of the experiment were inconsistent and did not correspond completely to the elevated plasma levels of CyPA. This implies that other inflammatory pathways were activated at the early stage of pathogenesis, which deserve further exploration. 3. The prolonged survival and the improvement of inflammatory organ infiltration in the CsA-treated mice were not apparent enough compared with the untreated group. This could be due to the following three reasons: First, IFNAR^−/−^ mice lack immune components that may largely influence the innate and adaptive immune responses after viral infection, and this could complicate the interpretation of the study results. Second, although we had previously shown that there was no significant change in the survival time and weight of mice in the 10 mg/kg CsA group, it is important to note that CsA is an immunosuppressant and the potential effects may be greater. Third, the SFTSV lethal model used in this study could result in insignificant efficacy in the CsA-treated group.

This study discussed the feasibility of CyPA inhibitor CsA as a treatment for SFTS. Other CyPA inhibitors with better efficacy and fewer side effects need to be explored as SFTS candidate drugs or therapies.

## Materials and methods

### Ethics statement

This study followed the principles of the Helsinki Declaration. Thirty SFTS patients diagnosed with SFTSV by a Nucleic Acid Quantitative Assay Kit (DAAN GENE, Guangzhou, China) were enrolled from the Department of Infectious Disease, First Affiliated Hospital of Nanjing Medical University, Nanjing, China, between October 2020 and November 2021. Twenty-nine healthy volunteers were also recruited into the study to serve as controls. Written informed consent was obtained from all participants, and the study was approved by the Institutional Review Board at First Affiliated Hospital of Nanjing Medical University. All animal-related experiments were conducted in animal biological safety level 2+ (BSL-2+) containment laboratories of Huadong Medical Institute of Biotechniques (HMIB), Nanjing, China. Mice experiments were approved by the HMIB ethics committee and conducted as per HMIB the ethical regulations for mice experiments.

### Cells and viruses

Vero and THP-1 cells were obtained from the cell bank of Chinese Academy of Sciences. Vero cells were maintained in Dulbecco’s Modified Eagle’s Medium (DMEM) supplemented with 10% fetal bovine serum (FBS; Gibco) and 1% antibiotics (penicillin and streptomycin; Gibco) in a humidified 5% CO_2_ atmosphere at 37°C. THP-1 cells were cultured in RPMI 1640 medium supplemented with 10% FBS and 1% antibiotics in a humidified 5% CO_2_ atmosphere at 37°C. PBMCs were derived from human peripheral blood isolated using Ficoll (Cytiva, Sweden). The isolated PBMCs were resuspended in DMEM medium supplemented with antibiotics and transferred into six-well plates (Corning, United States) at a density of 2 × 10^7^ cells/well. After 4 h, the supernatant was discarded, and PBMCs were cultured in RPMI1640 medium containing 10% FBS and 1% penicillin and streptomycin.

The Chinese SFTSV strain JS14 was provided by the Center for Diseases Prevention and Control of Jiangsu Province. All SFTS viruses were propagated in Vero cells and stored at −80°C until use. The infectious doses of SFTSV in plasma and supernatants were determined by the DAAN SFTSV Nucleic Acid Quantitative Assay Kit (DA 0340). Experiments involving infectious SFTSV were performed in BSL-2+ containment laboratories of HMIB, and operations followed institutional biosafety procedures.

### Compounds

CsA (HY-B0579), hrCyPA (HY-P70047), AC-73 (HY-122214), PEG 300 (HY-Y0873), and Tween 80 (HY-Y1891) were purchased from MedChemExpress compound library (MCE). CsA, hrCyPA, and AC-73 were dissolved in phosphate-buffered saline (PBS) and used for *in vitro* experiments. Ten milligrams of CsA powder was dissolved in 200 μl dimethyl sulfoxide (DMSO) and then diluted with 40% PEG 300, 5% Tween 80, and 53% PBS to a working solution of 1 mg/ml for *in vivo* experiments.

### Lentiviral construct

The shRNA lentiviral vector for CyPA was synthesized by Hanbio Biotechnology (Shanghai, China) using the following sequence: CGTTTGAGT-TAAGAGTGTTGATGTA. In THP-1 cells, sh-CyPA cells were successfully constructed according to the manufacturer’s instructions.

### RNA extraction and reverse transcription-quantitative PCR (qRT-PCR)

Total RNA was isolated using the TRIzol method (Invitrogen, United States) and first-strand cDNA synthesis was performed using PrimeScript RT Master Mix (Takara, Japan). Real-time PCR was performed on an ABI QuantStudio 5 sequence detection system using TB Green Premix Ex Taq (Takara, Japan). The qPCR primers are shown in Table 1. GAPDH was used as an endogenous control for mRNA relative expression.

**Table 1:** Real-time PCR primer pairs.

Primer pair	Sequence (5′ to 3′)
SFTSV	F:5′-TGGAATGCAGACCTTGTGGG-3′
	R:5′-GCCTGTCATGCAAGAATAACATC-3′
IL-6	F:5′-ACCCCCAGGAGAAGATTCCA-3′
	R:5′-TCACCAGGCAAGTCTCCTCA-3′
IL-1β	F:5′-ATGATGGCTTATTACAGTGGCAA-3′
	R:5′-GTCGGAGATTCGTAGCTGGA-3′
TNF-α	F:5′-GCTGCACTTTGGAGTGATCG-3′
	R:5′-TATCTCTCAGCTCCACGCCA-3′
GAPDH	F:5′-ACAACTTTGGTATCGTGGAAGG-3′
	R:5′-GCCATCACGCCACAGTTTC-3′

F, forward; R, reverse.

### Western Blot

Total cellular protein extraction was done according to the manufacturer’s instructions (BestBio, China). The proteins were denatured at 95°C for 5 min, separated by SDS-PAGE using 12% polyacrylamide gels, and then transferred to a nitrocellulose membrane (Sangon Biotech, China). After blocking with 5% Bovine Serum Albumin (BSA; Roche) in Tris-buffered Saline (TBS), the membranes were examined with primary antibodies and the corresponding horseradish peroxidase-conjugated secondary antibodies. Washing was done thoroughly between each step using TBS-T (TBS supplemented with 0.1% Tween 20, pH 7.4). Protein signals were detected with enhanced chemiluminescence kit (ECL; Tanon) and visualized using the chemiluminescent imaging system (Tanon 5,200, China). Target bands were blotted with various primary antibodies (CyPA and CD147, ProteinTech Group; phospho-ERK1/2, phospho-p38, phospho-JNK, phospho-NF-κB, ERK1/2, p38, JNK, and NF-κB, Cell Signaling Technology; GAPDH, Affinity). The anti-rabbit and anti-mouse IgG antibodies (Cell Signaling Technology; United States) were used as the secondary antibodies. The intensities of specific protein bands were quantified using ImageJ analysis software and corrected for the intensity of the respective GAPDH band.

### Enzyme-linked immunosorbent assay

The levels of CyPA (CUSABIO, China) and pro-inflammatory cytokines (IL6, IL-1β, and TNFα; R&D systems, United States) in plasma and cell supernatants were determined using ELISA.

### Animal experiments

IFNAR^−/−^ C57BL/6 mice were identified and purchased from Cyagen (Cyagen Biosciences Inc.). All mice were bred and maintained in an environmentally controlled and specific-pathogen-free (SPF) animal facility at HMIB.

Six-to eight-week-old mice were randomly divided into three groups of three mice each. On day 0, the untreated and CsA-treated mice were subcutaneously inoculated with 100 μl of 1.0 × 10^6^ TCID_50_/ml virus solution, while the mock mice were injected subcutaneously with 100 μl of DMEM. For the CsA-treated mice group, 10 mg/ml CsA was given 1 h, 1 day, 2 days, 3 days, 4 days, and 5 days post-infection and continued for 5 days. Blood samples were obtained by tail vein puncture on days 1, 3, and 5 of the experiment to measure viral RNA, CyPA, and pro-inflammatory cytokines in plasma.

### Histopathology and immunohistochemistry

Liver, spleen, lung, kidney, and brain tissues were collected from each group of mice, embedded in paraffin, sectioned, and stained with hematoxylin and eosin (H&E). Immunohistochemistry (IHC) was used to detect the location and expression of SFTSV nucleoprotein (NP) in the paraffin-embedded sections. A monoclonal antibody against SFTSV NP derived from mice (Cambridge biologics, USA) was used as the primary antibody for IHC. After deparaffinization in dimethylbenzene and rehydration in the gradient ethanol, 10 mM citric acid (pH 6.0) was added, and the sections were heated in a microwave oven for antigen retrieval. Staining was performed according to the manufacturer’s instructions: sections were incubated with the primary antibody overnight at 4°C and then incubated with the secondary antibody. Color development was performed using avidin–biotin complex (ABC; Vector Laboratories, United States) and 3,3-N-Diaminobenzidine Tetrahydrochloride (DAB; Beyotime, China). Sections were counterstained with hematoxylin.

### Statistical analysis

An unpaired two-tailed Student’s *t*-test was used to assess differences between the two groups. Data sets involving more than two groups were evaluated using the Newman–Keuls test. A value of *p* of 0.05 was considered statistically significant.

## Data availability statement

The original contributions presented in the study are included in the article/Supplementary material, further inquiries can be directed to the corresponding authors.

## Ethics statement

The studies involving human participants were reviewed and approved by Ethics Committee of Jiangsu Provincial People's Hospital. The patients/participants provided their written informed consent to participate in this study. The animal study was reviewed and approved by the Huadong Medical Institute of Biotechniques.

## Author contributions

HH: writing-original draft. HH, KO, YZ, QZ, JZ, XS, DL: validation and formal analysis. KJ: methodology and project administration. ZJ: formal analysis. ZY: methodology. NH and YD: data curation. YH, HL, CHW, and CYW: writing—review and editing. JZ and JL: funding acquisition and supervision. All authors contributed to the article and approved to the submitted version.

## Funding

The work was supported by the National Natural Science Foundation of China (no.: 81871242) and Surface Project of Jiangsu Health Commission (no.: H2018019).

## Conflict of interest

The authors declare that the research was conducted in the absence of any commercial or financial relationships that could be construed as a potential conflict of interest.

## Publisher’s note

All claims expressed in this article are solely those of the authors and do not necessarily represent those of their affiliated organizations, or those of the publisher, the editors and the reviewers. Any product that may be evaluated in this article, or claim that may be made by its manufacturer, is not guaranteed or endorsed by the publisher.
