# Supplementary material for: Cyclophilin A causes severe fever with thrombocytopenia syndrome virus-induced cytokine storm by regulating mitogen-activated protein kinase pathway
Source: Front Microbiol. 2022 Dec 1;13:1046176. doi: 10.3389/fmicb.2022.1046176 (PMC9768865; doi:10.3389/fmicb.2022.1046176)
Supplement: Supplementary file 1 [file Data_Sheet_1.PDF]

Supplementary Appendix

Supplementary figures

Supplementary figure S1

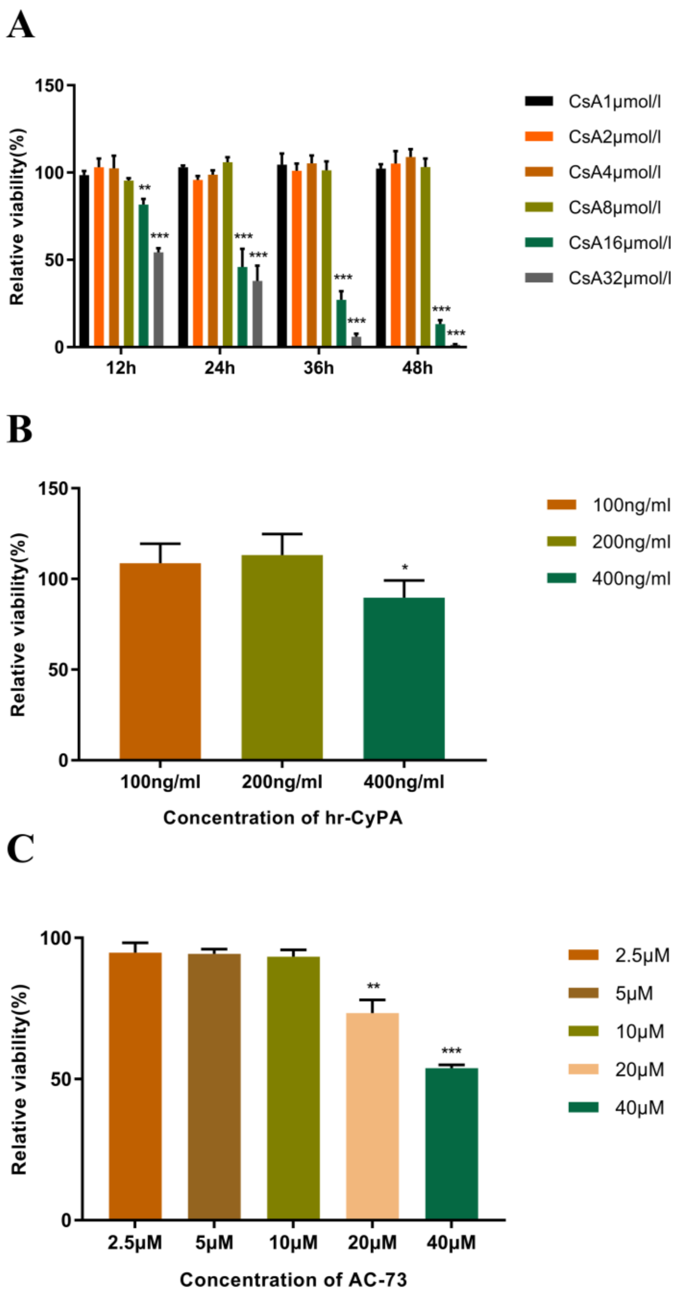

Figure S1. CCK8 assay showing the working concentration of CsA (A), hr-CyPA (B), AC-73 (C).

Supplementary figure S2

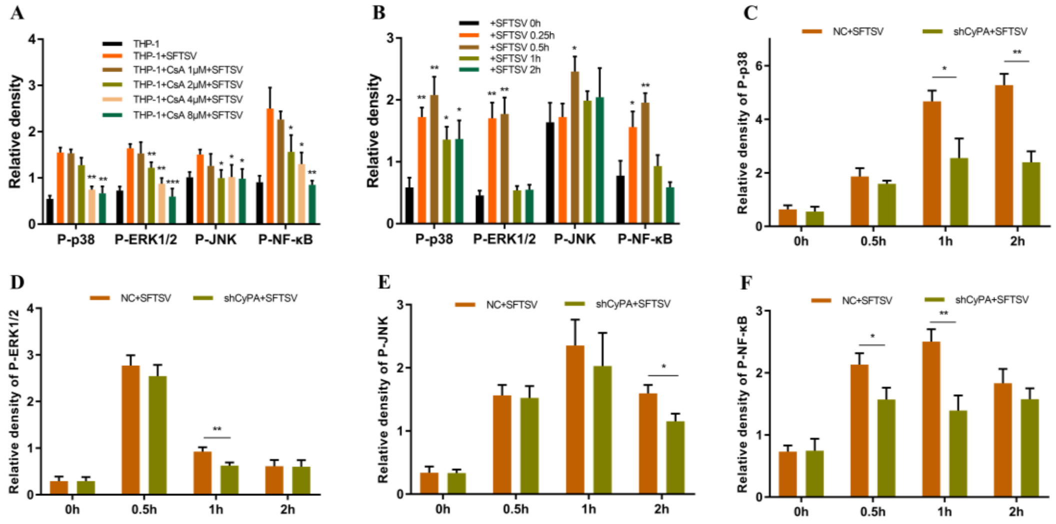

Figure S2. Key proteins of Western blot quantified by image J. (A) Gray value analysis of Figure 2C. (B) Gray value analysis of Figure 3D. (C-F) Gray value analysis of Figure 3E.

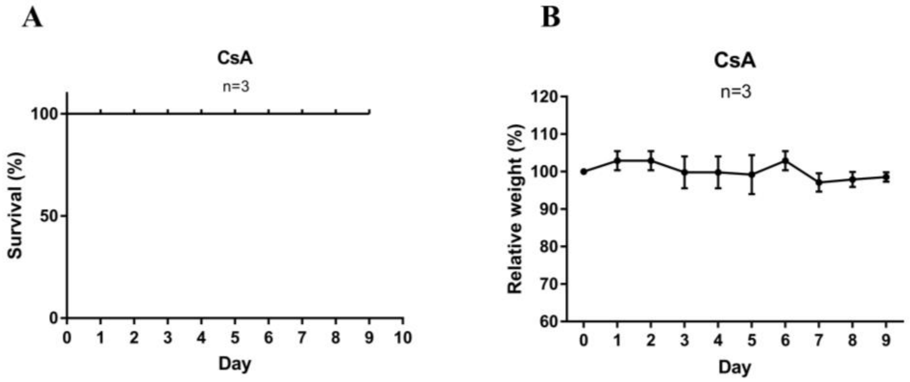

Figure S3. CsA at 10 mg/kg/day had no significant toxic effect on IFNAR$^{-/-}$ mice. (A) Three mice were treated with 10mg/kg/day CsA for five consecutive days. Survival curves were determined using GraphPad Prism7. (B) The relative weight is shown as means with standard deviations.

Supplementary methods

Cell counting kit-8 (CCK8) assay. The CCK8 assay kit (DOJINDO., Japan) was used to examine cell viability. THP-1 cells were inoculated in 96-well plates at a density of 2×10^4 cells/100 μL/well and compound solutions were added to each well at different concentrations (CsA 1, 2, 4, 8, 16 and 32 μM; hr-CyPA 100, 200 and 400 ng/mL; AC-73 2.5, 5, 10, 20 and 40 μM). Control cells were treated with 100 μL basal medium. All the THP-1 cells were cultured in a constant temperature incubator with 5% CO2 at 37 °C. THP-1 cells treated with different concentrations of

CsA, hr-CyPA and AC-73 were cultured for 24 h, 12 h and 4 h, respectively. Subsequently, 10 μL CCK8 solution was added to each well and cells were cultured in an incubator for another 4 h. Finally, absorbance was detected at 450 nm. The data were analyzed using GraphPad.
