# Supplementary material for: Cyclophilin A causes severe fever with thrombocytopenia syndrome virus-induced cytokine storm by regulating mitogen-activated protein kinase pathway
Source: Front Microbiol. 2022 Dec 1;13:1046176. doi: 10.3389/fmicb.2022.1046176 (PMC9768865; doi:10.3389/fmicb.2022.1046176)

Histopathological examinations of the tissues collected from the untreated and CsA-treated mice.

1. Mock-liver.


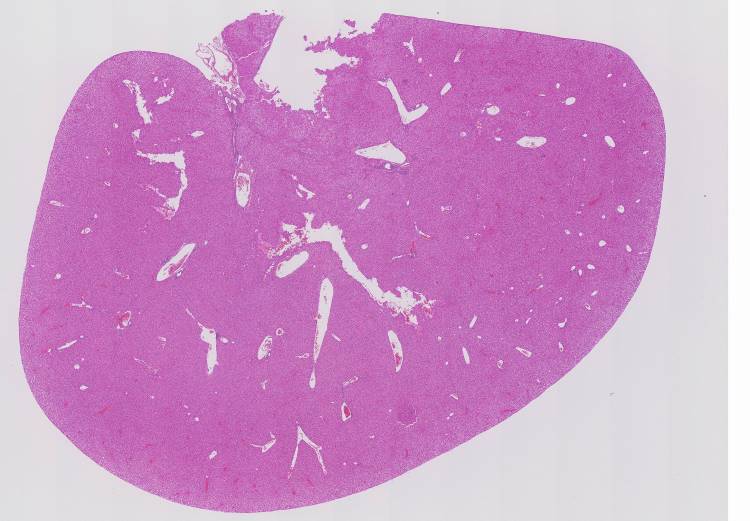


1. Mock-spleen


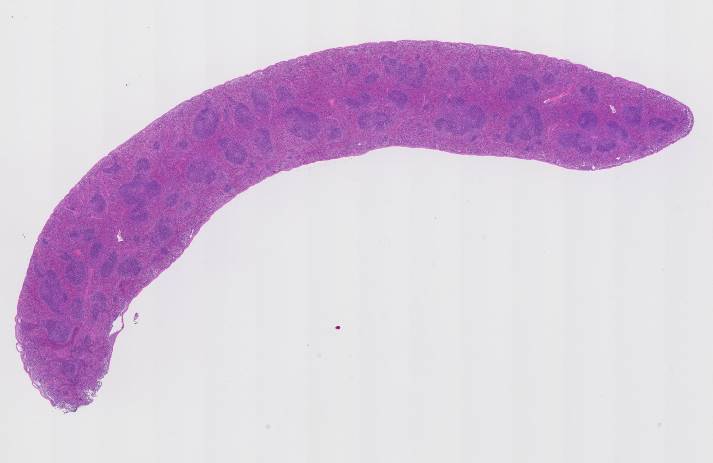


1. Mock-lung


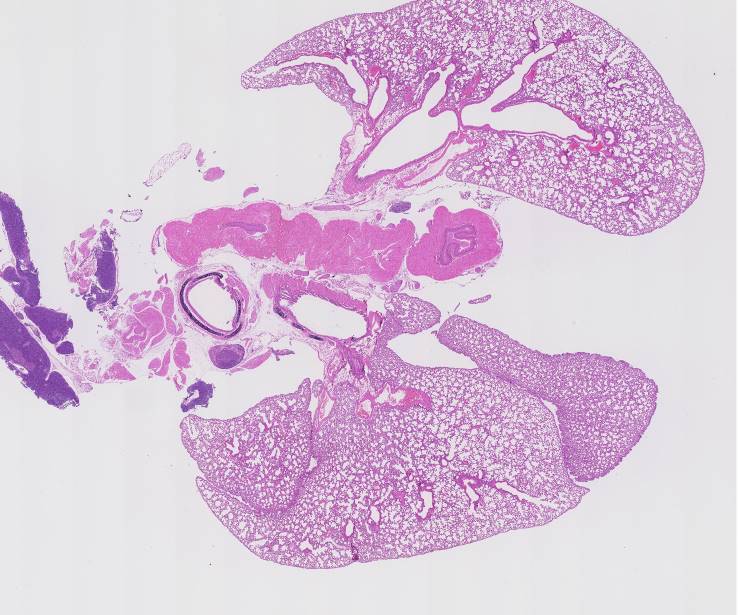


1. Mock-kidney


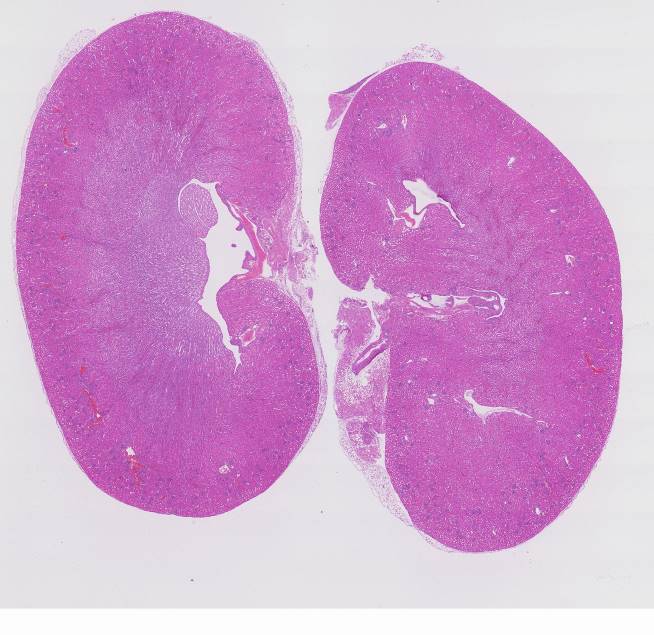


1. Mock-brain


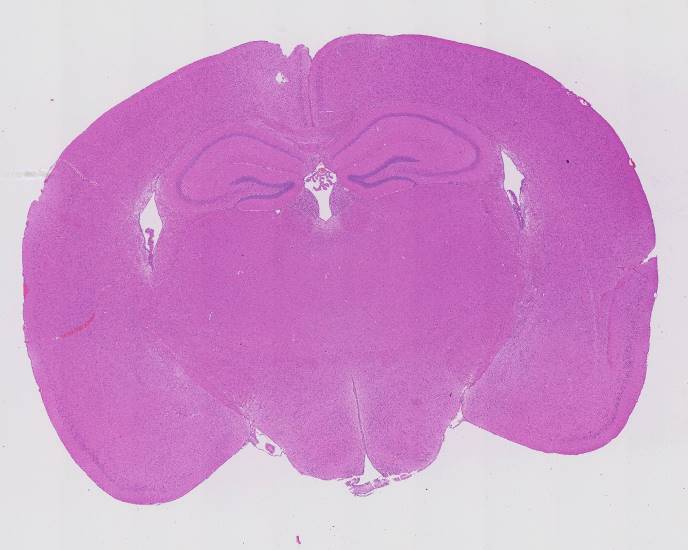


1. Untreated-liver


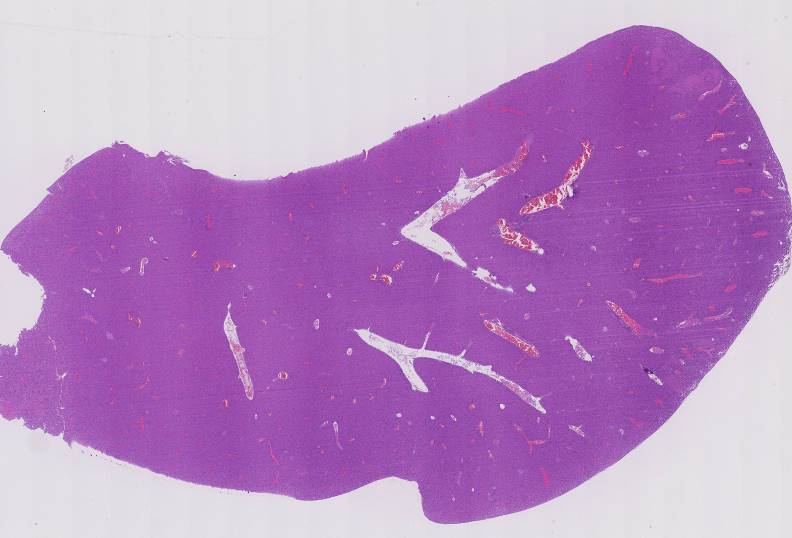


1. Untreated-spleen


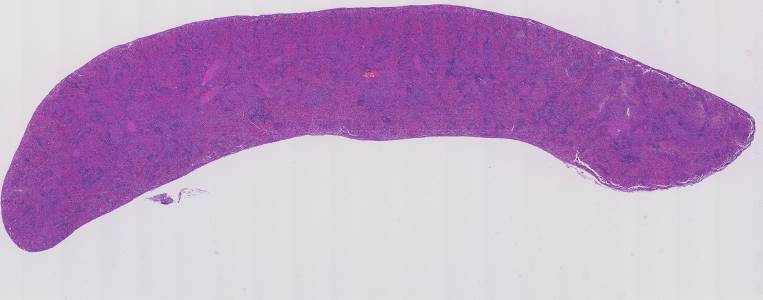


1. Untreated-lung


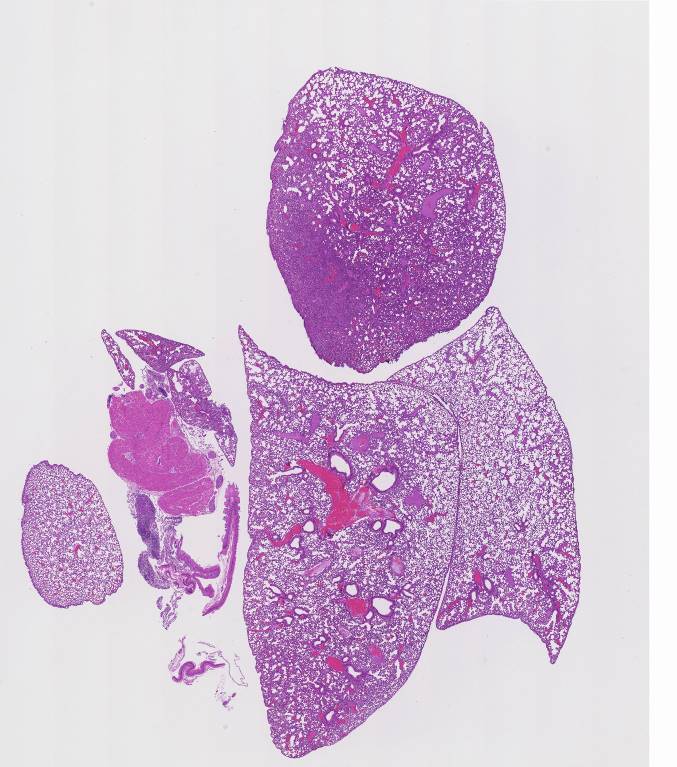


1. Untreated-kidney


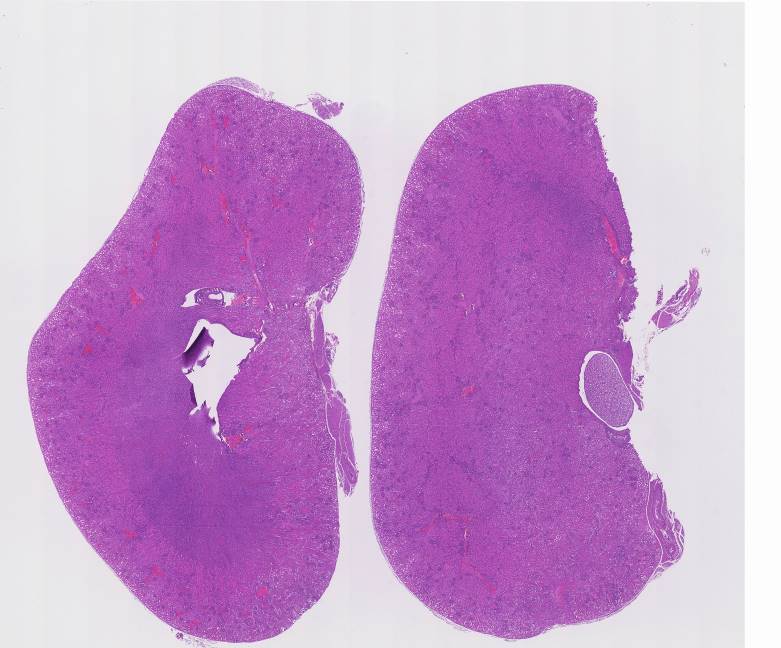


1. Untreated-brain


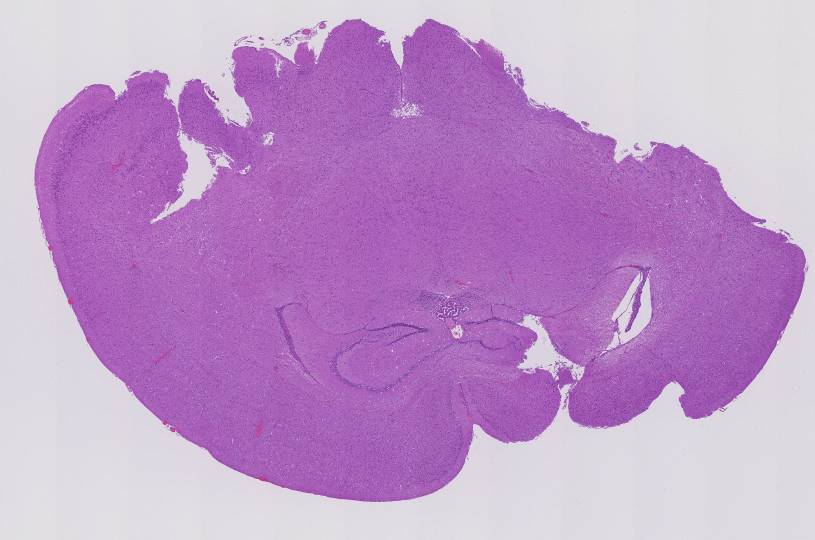


1. CsA-treated-liver


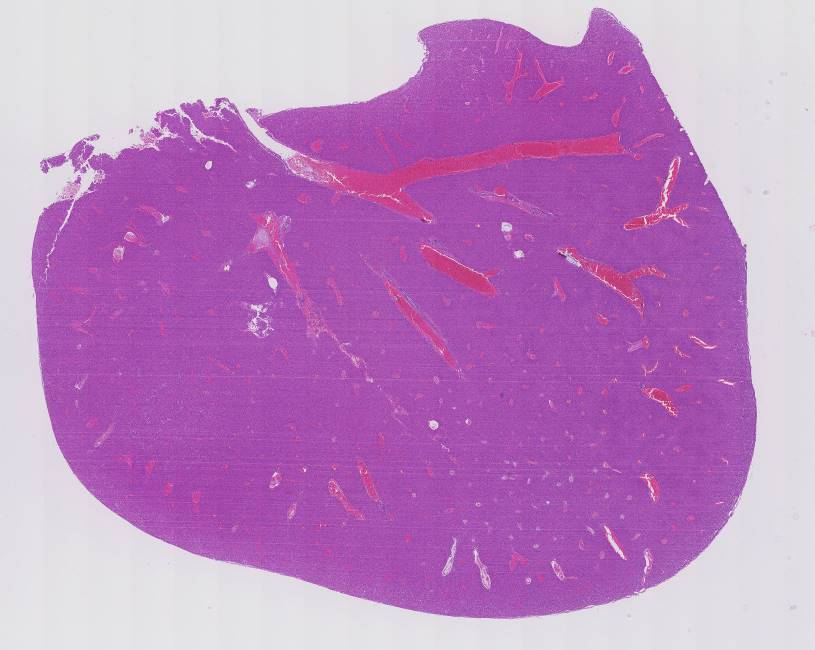


1. CsA-treated-spleen


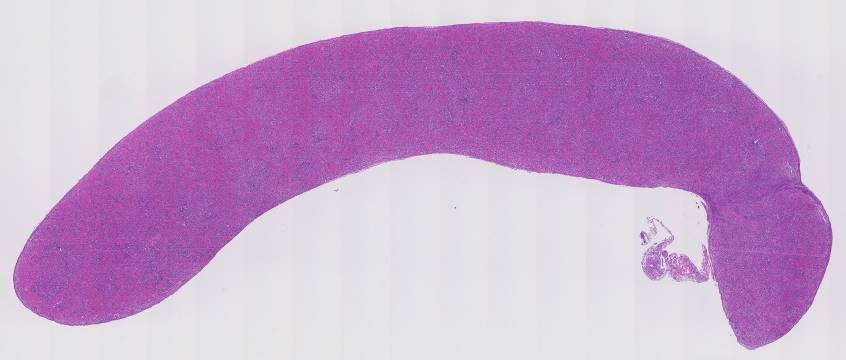


1. CsA-treated-lung


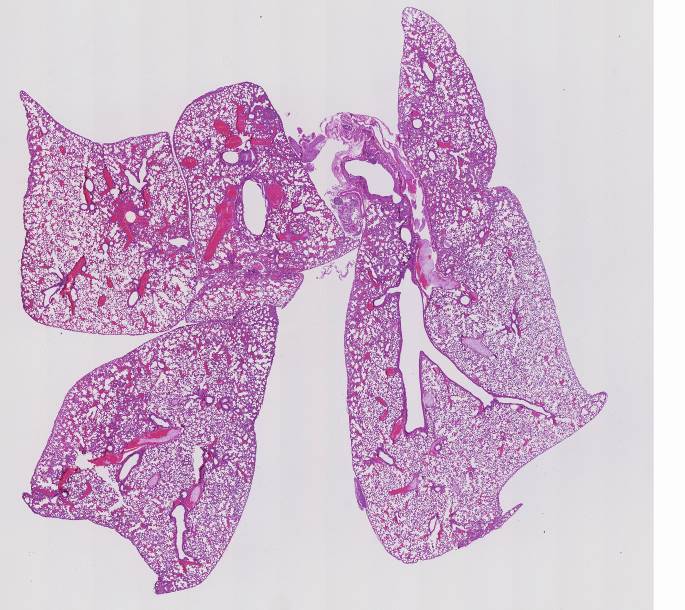


1. CsA-treated-kidney


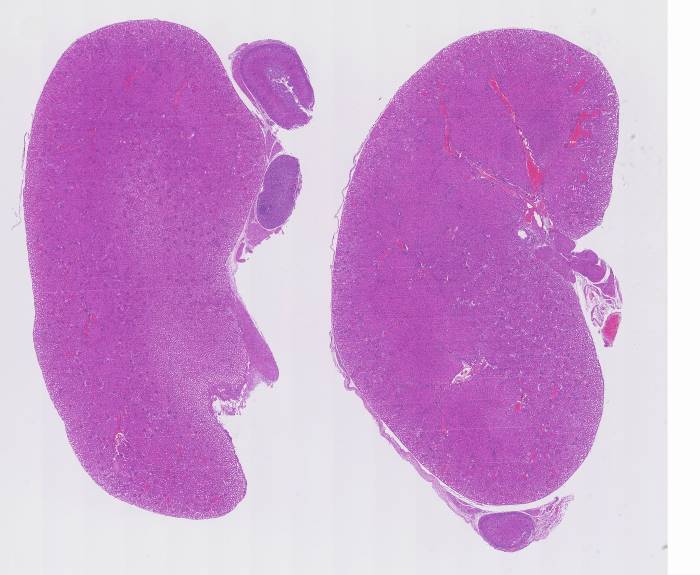


1. CsA-treated-brain


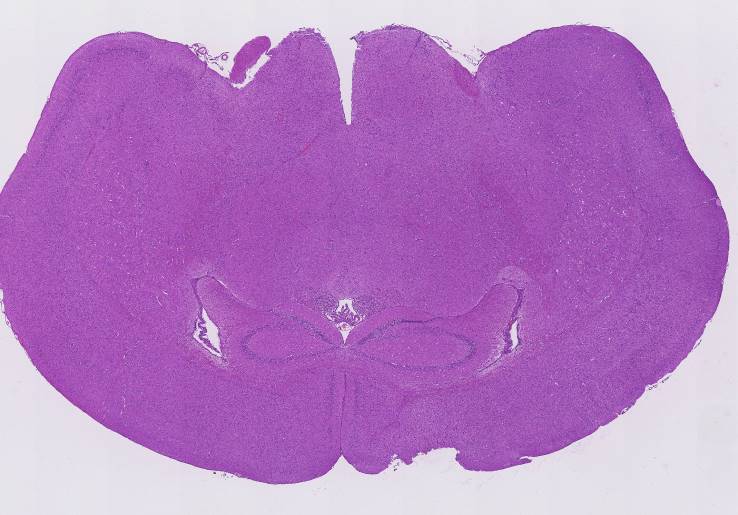


Figure2. Immunohistochemical examinations of the tissues collected from the untreated and CsA-treated mice.

1. Mock-liver.


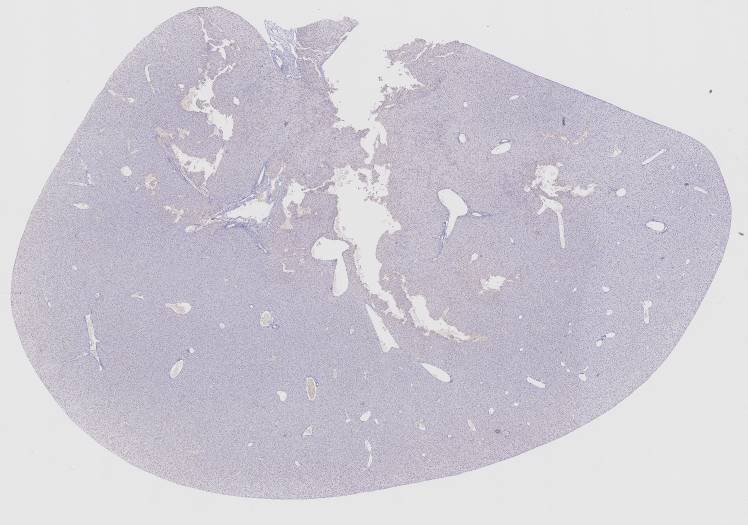


1. Mock-spleen


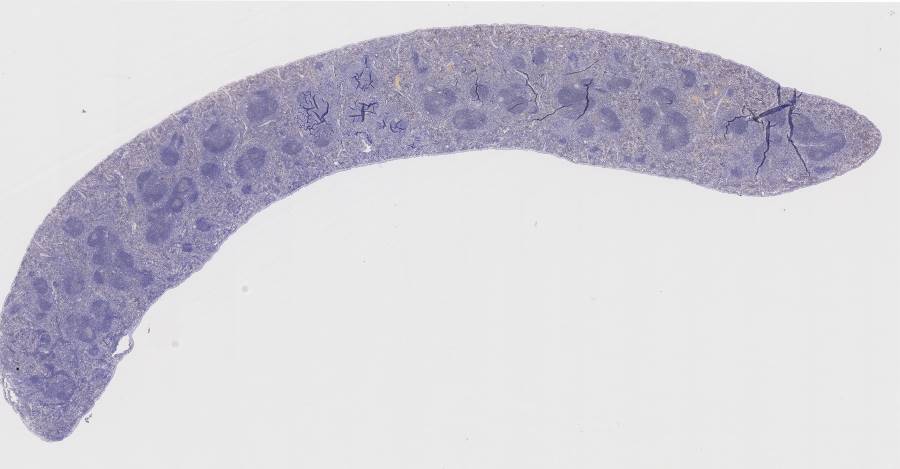


1. Mock-lung


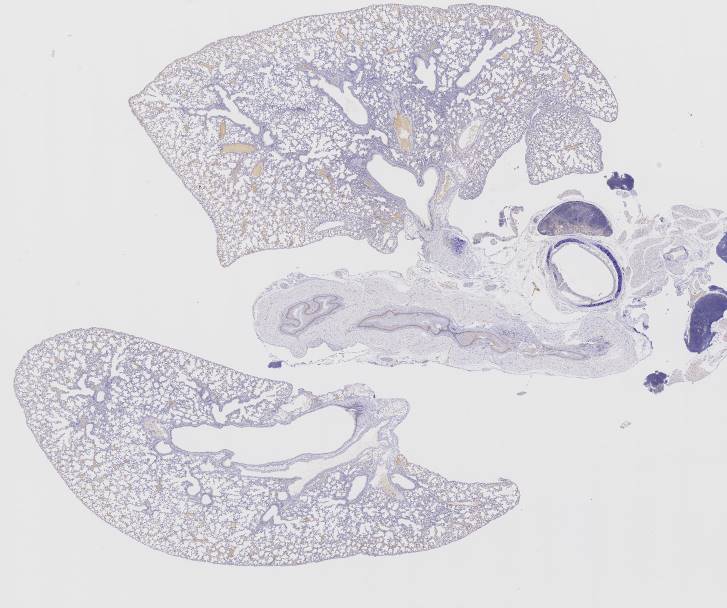


1. Mock-kidney


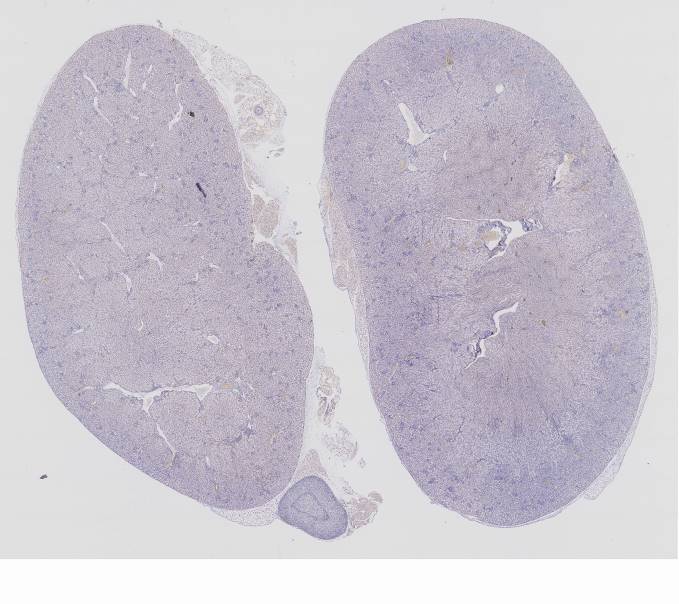


1. Mock-brain


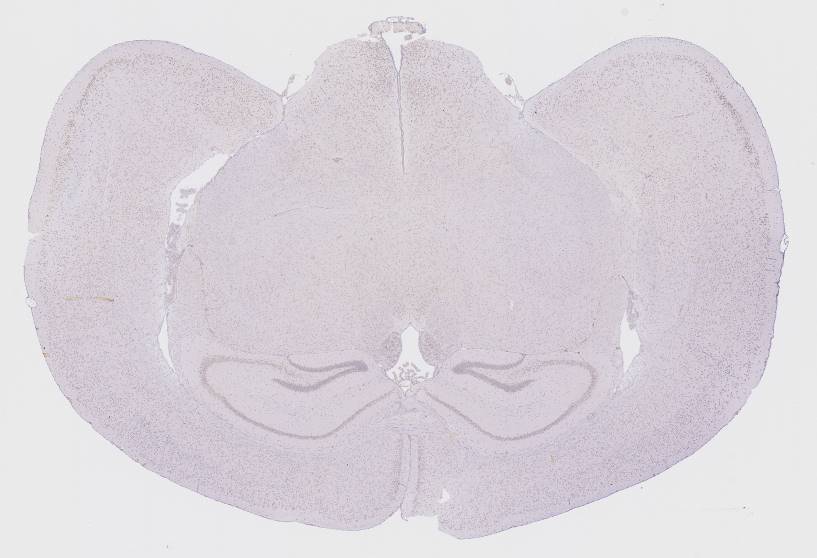


1. Untreated-liver.


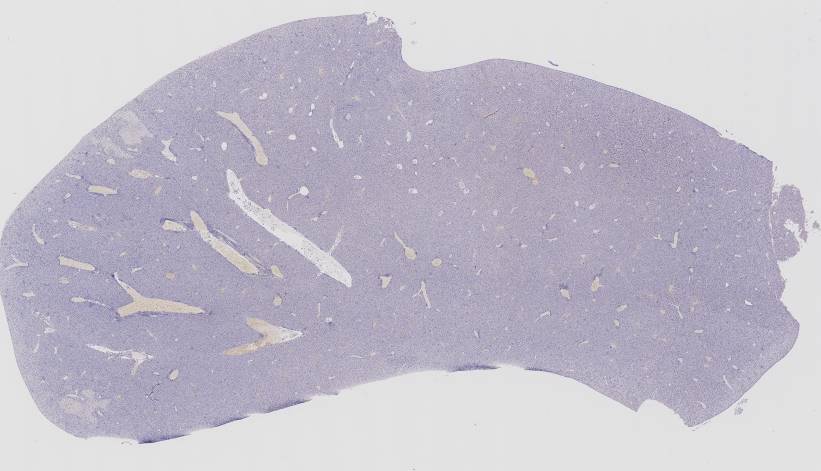


1. Untreated-spleen


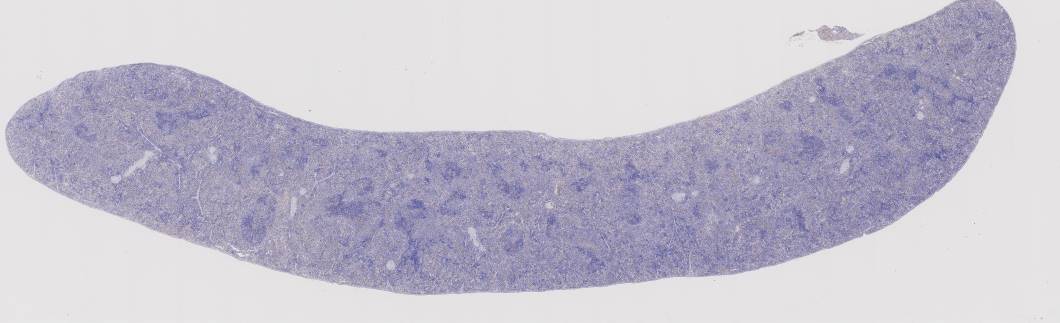


1. Untreated-lung


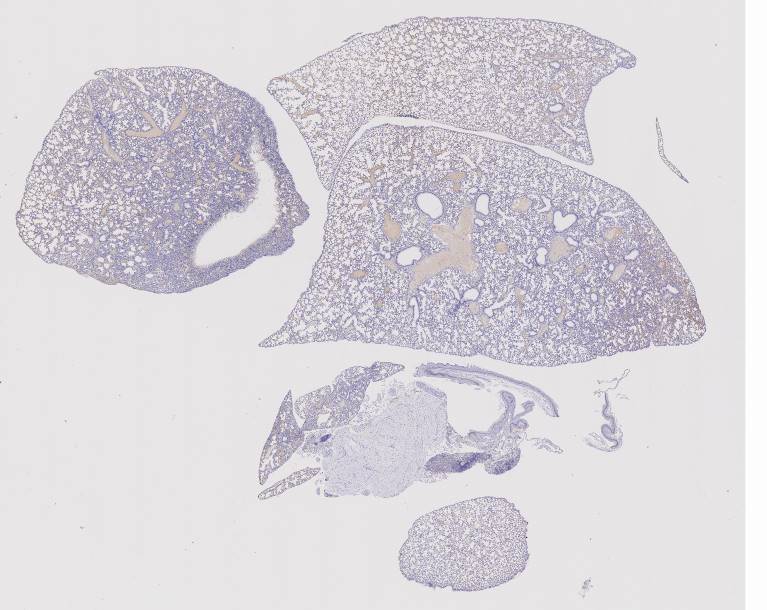


1. Untreated-kidney


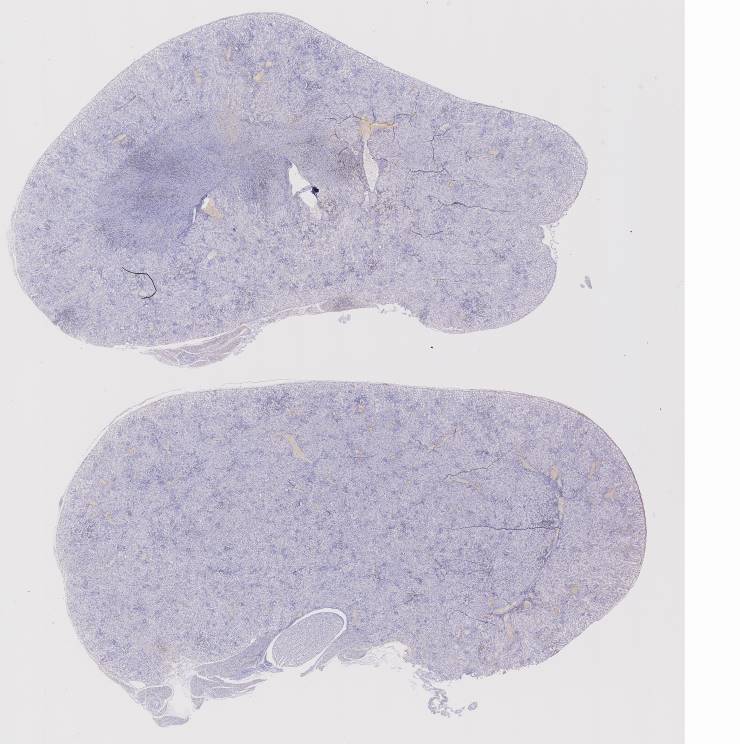


1. Untreated-brain


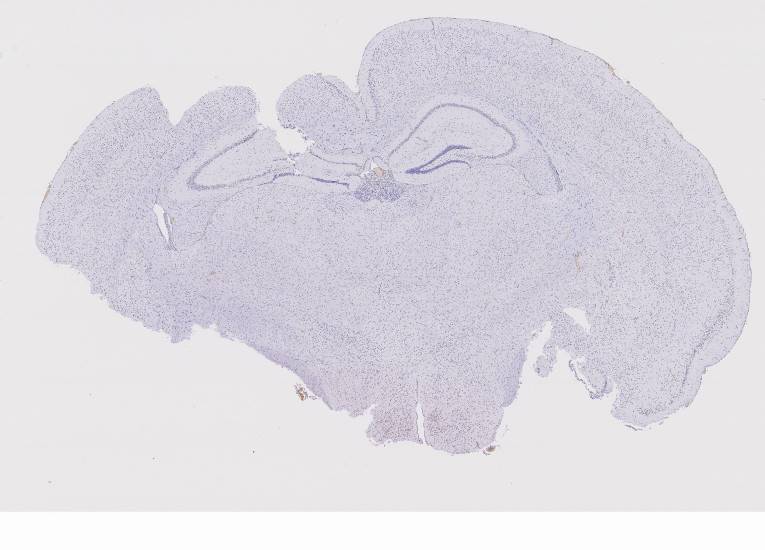


1. CsA-treated-liver


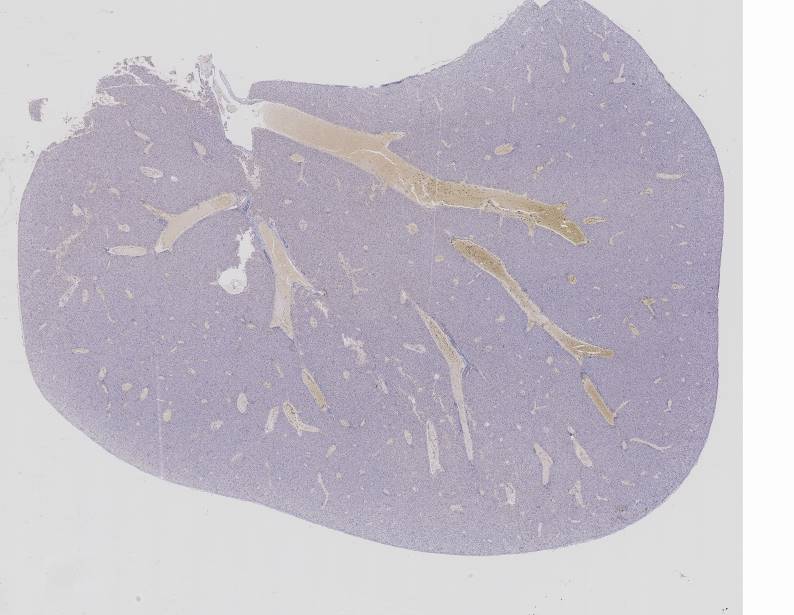


1. CsA-treated-spleen


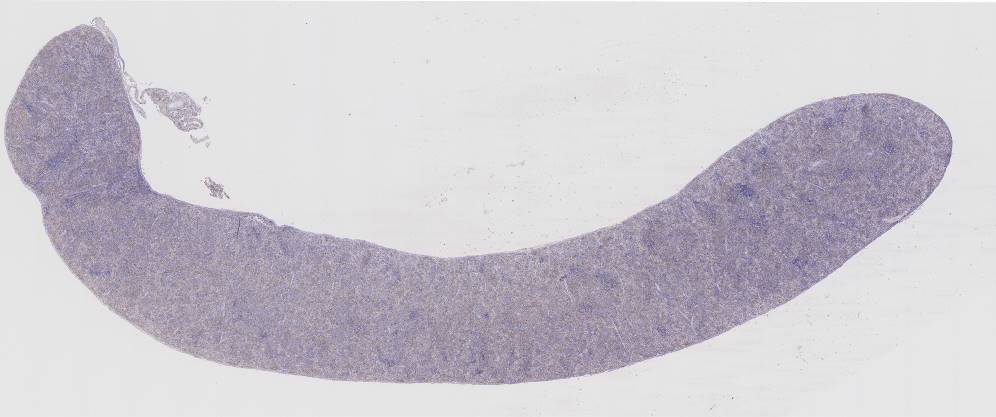


1. CsA-treated-lung


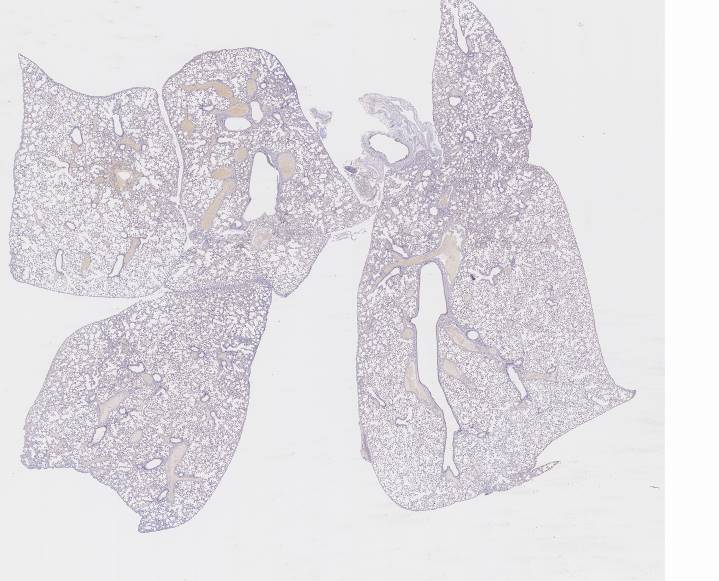


1. CsA-treated-kidney


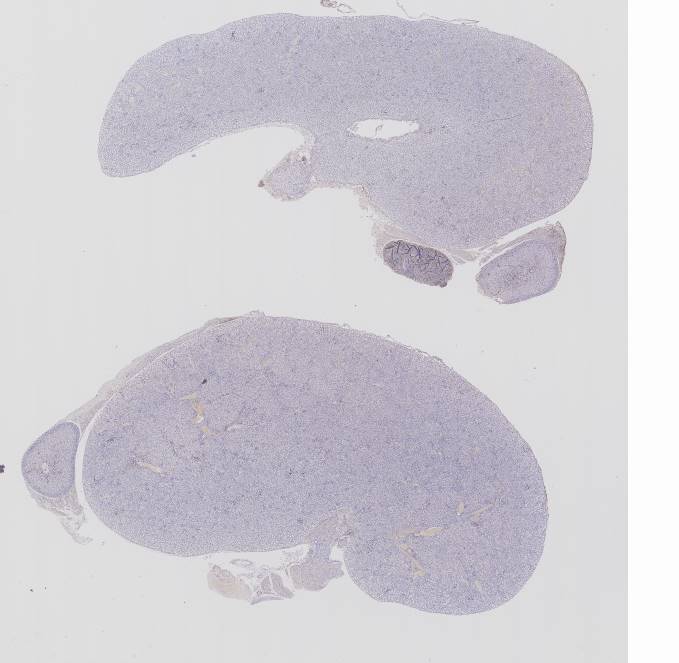


1. CsA-treated-brain


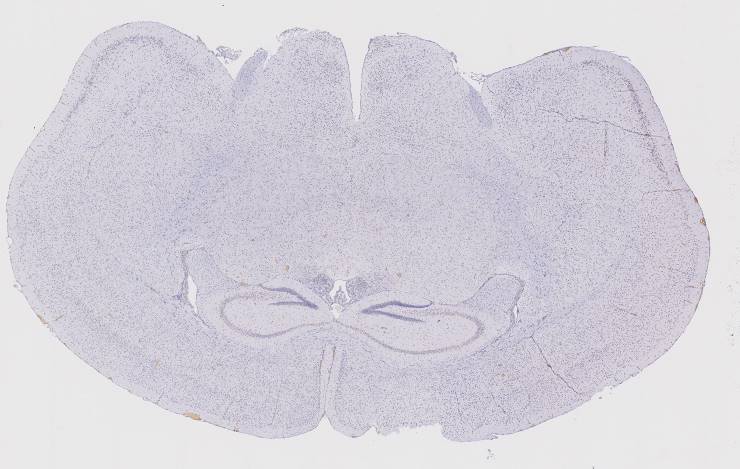


Figure3. Original scanned picture of WB.

1. (Figure2C of article)

图2-C-1-Pp38


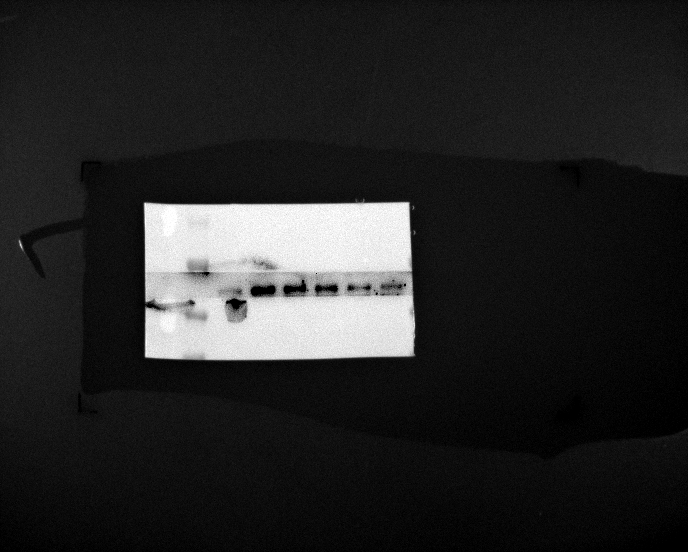


图2-C-2-p38


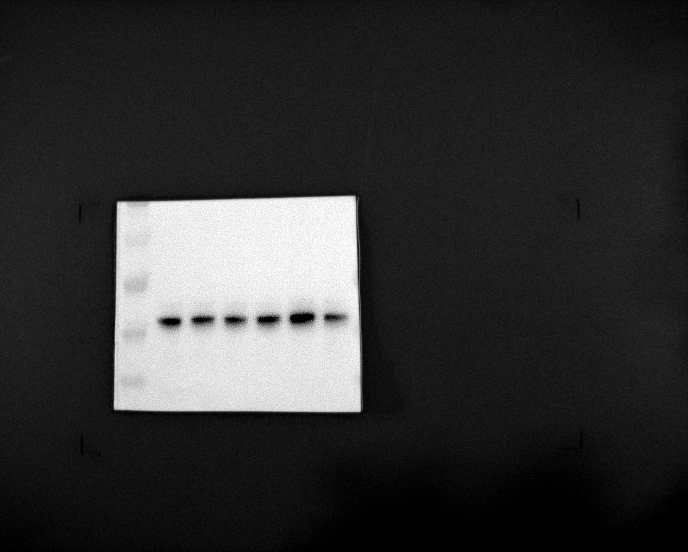


图2-C-3-PERK


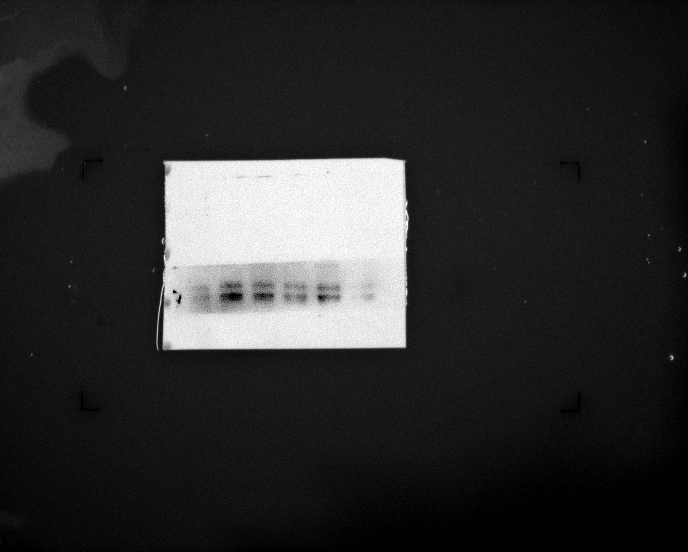


图2-C-4-ERK


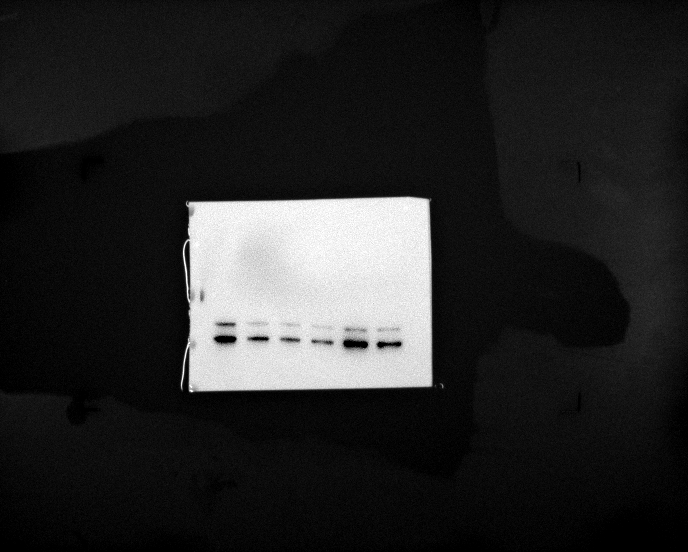


图2-C-5-PJNK


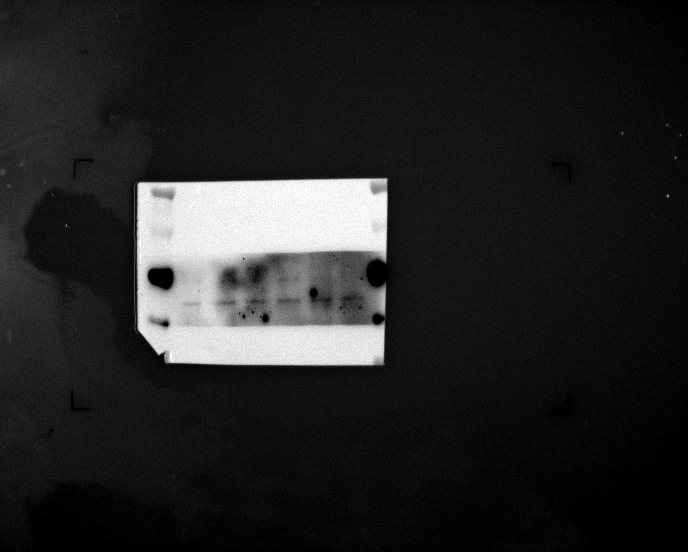


图2-C-6-JNK


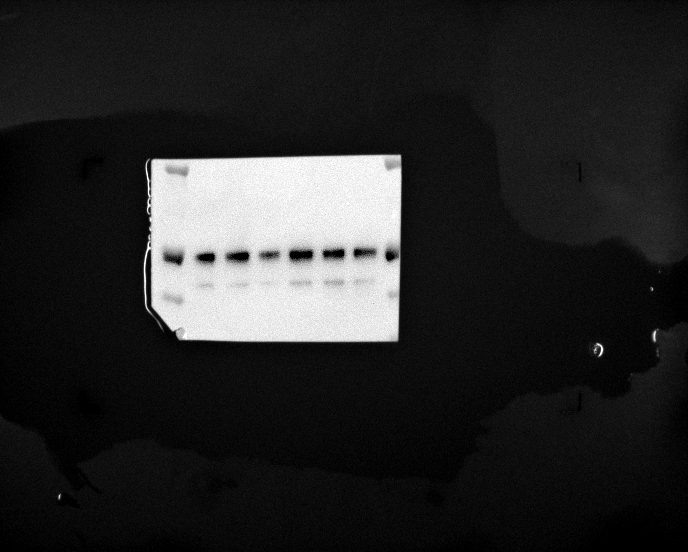


图2-C-7-PNF-κB


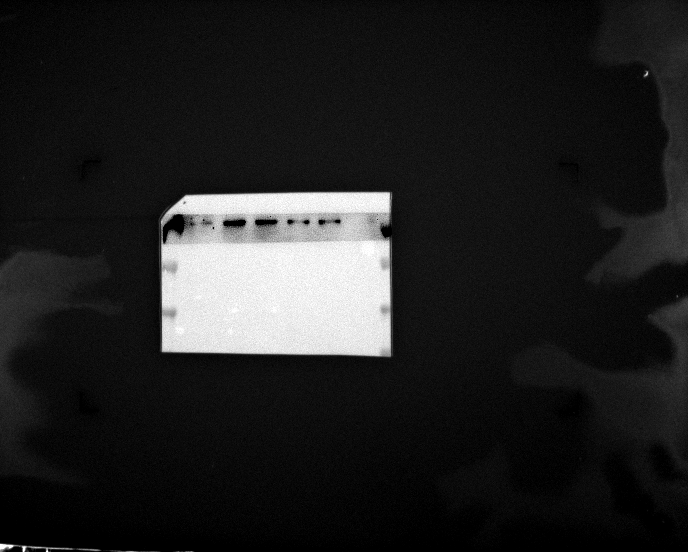


图2-C-8-NF-κB


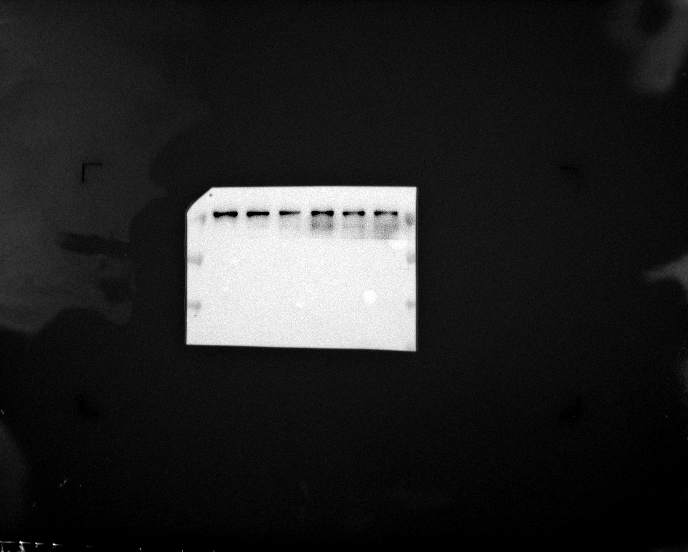


图2-C-9-GAPDH


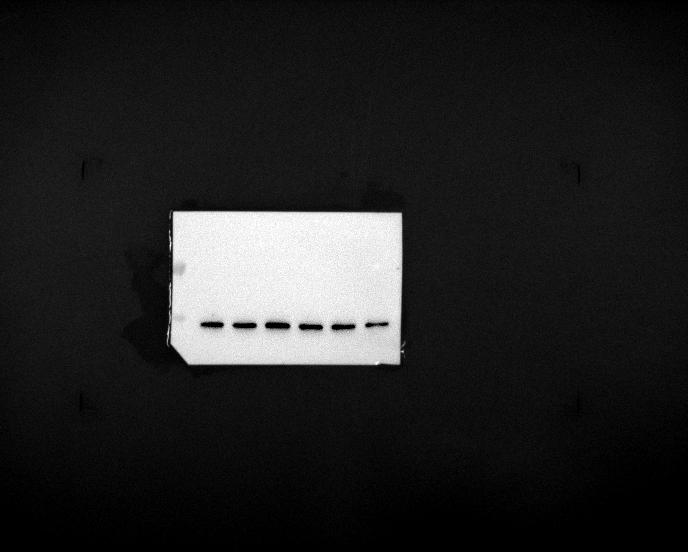


1. (Figure3B of article)

图3-B-1-CyPA


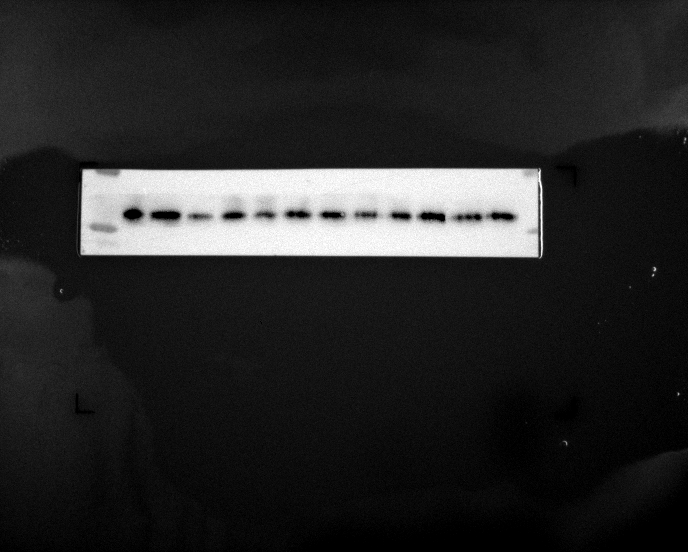


图3-B-2-GAPDH


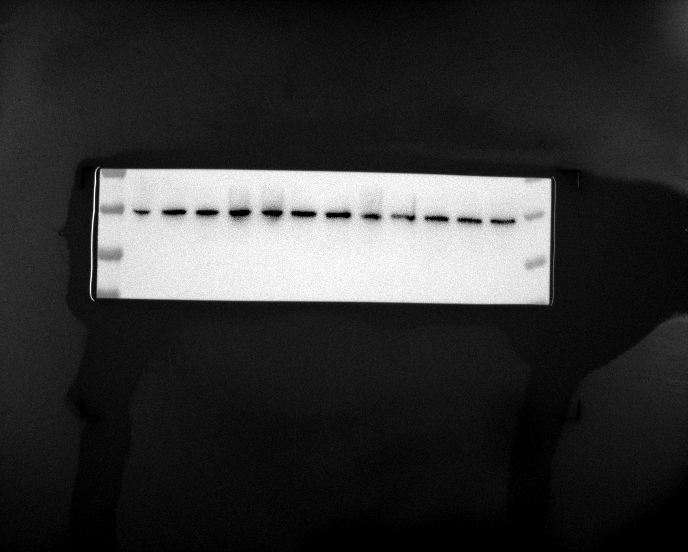


1. ( Figure3D of article)

3D-1-Pp38


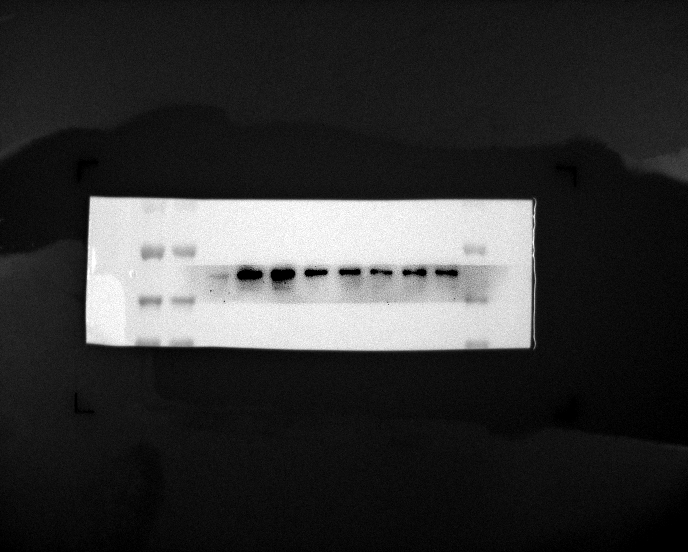


3D-2-p38


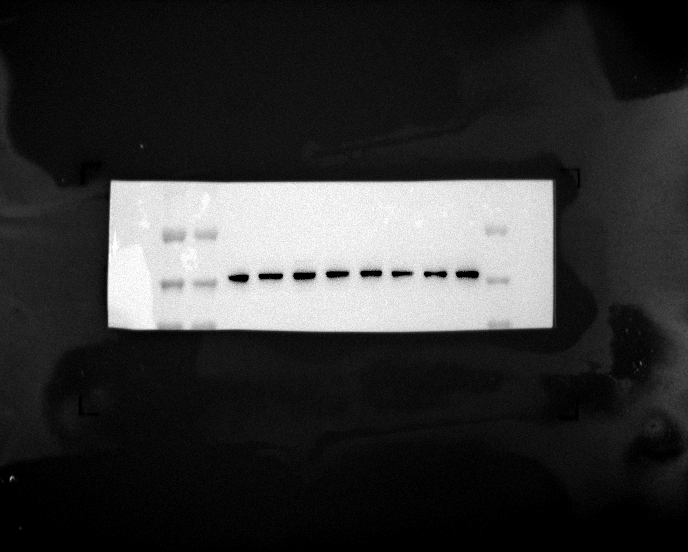


3D-3-PERK


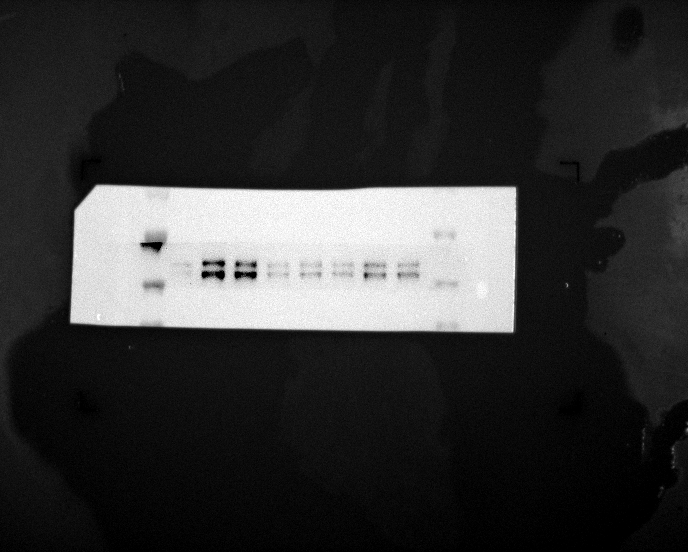


3D-4-ERK


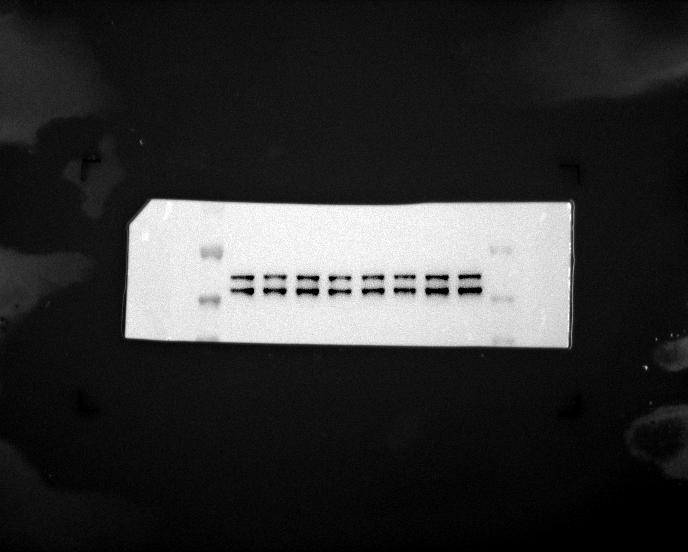


3D-5-PJNK


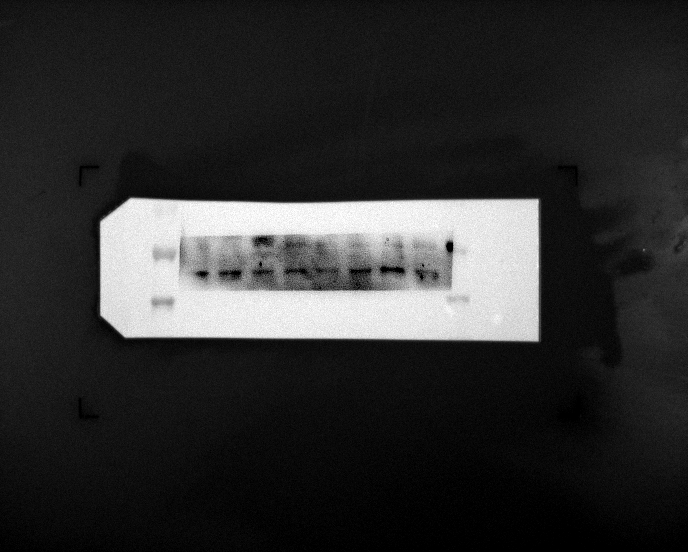


3D-6-JNK


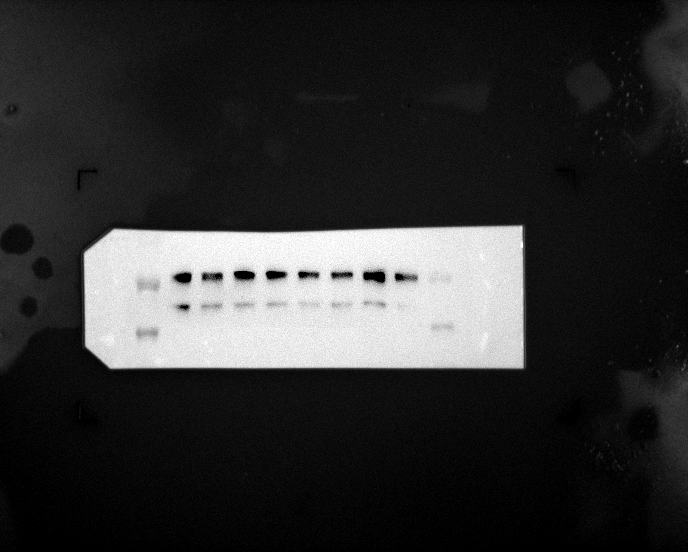


3D-7-PNF-κB


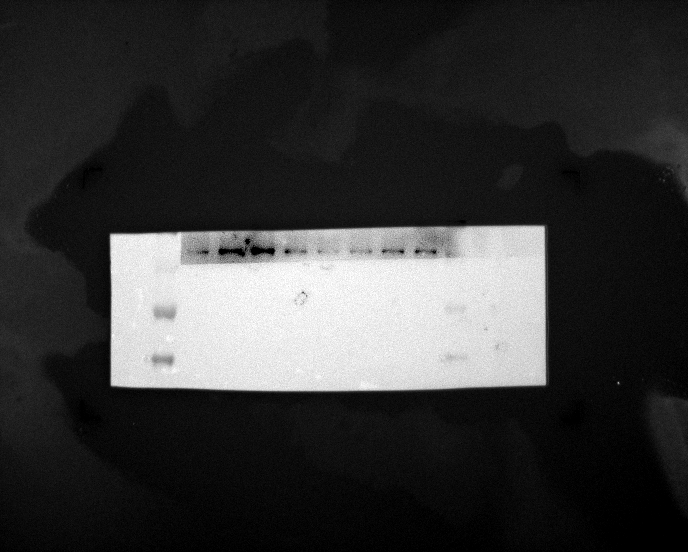


3D-8-NF-κB


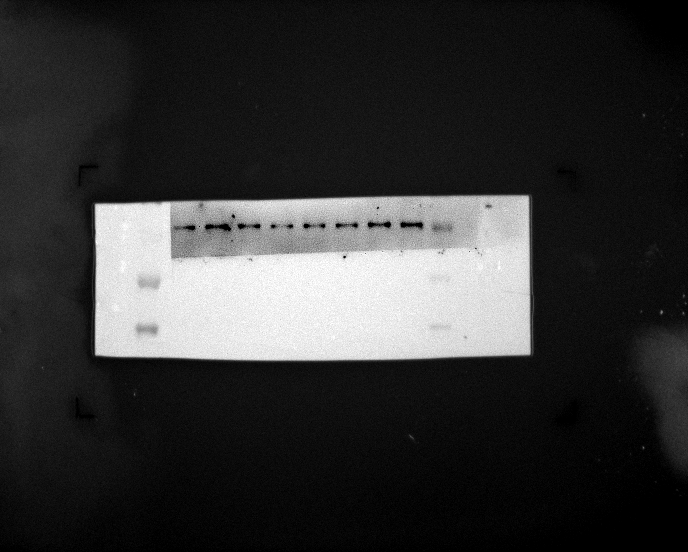


3D-9-GAPDH


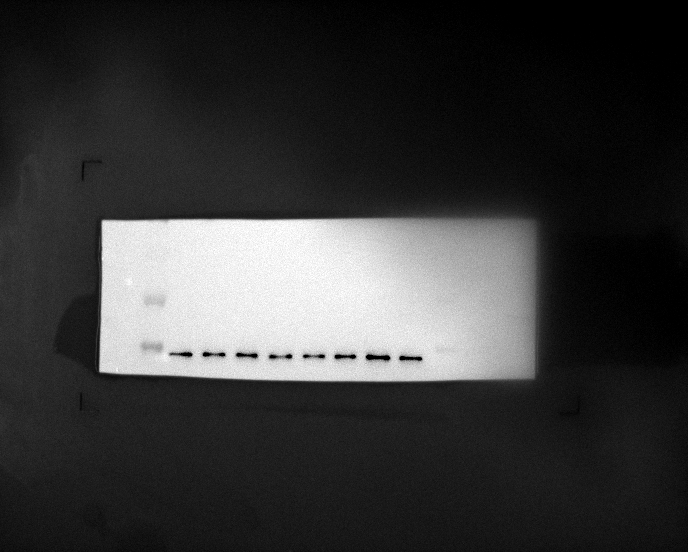


1. ( Figure3E of article)

3E-1-Pp38


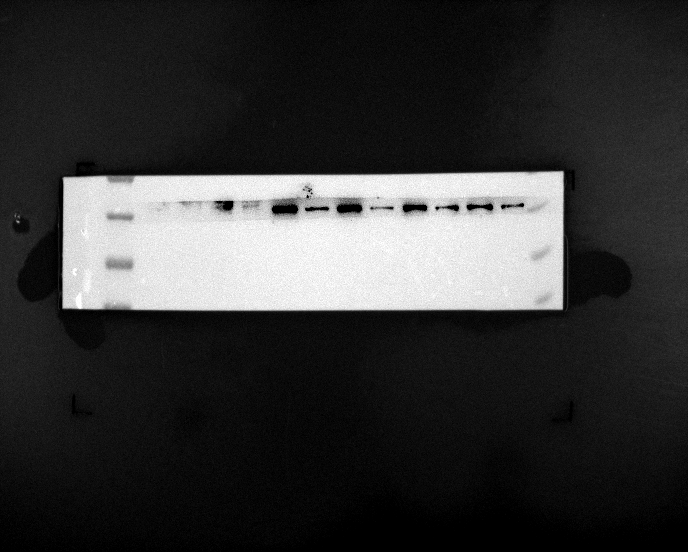


3E-2-p38


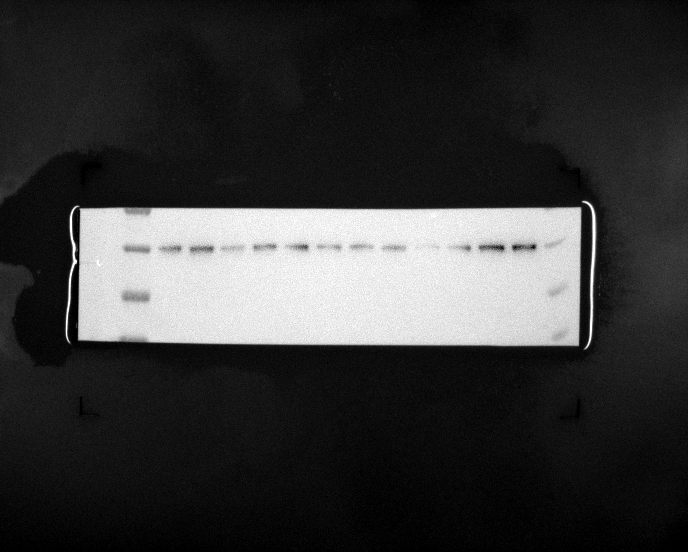


3E-3-PERK


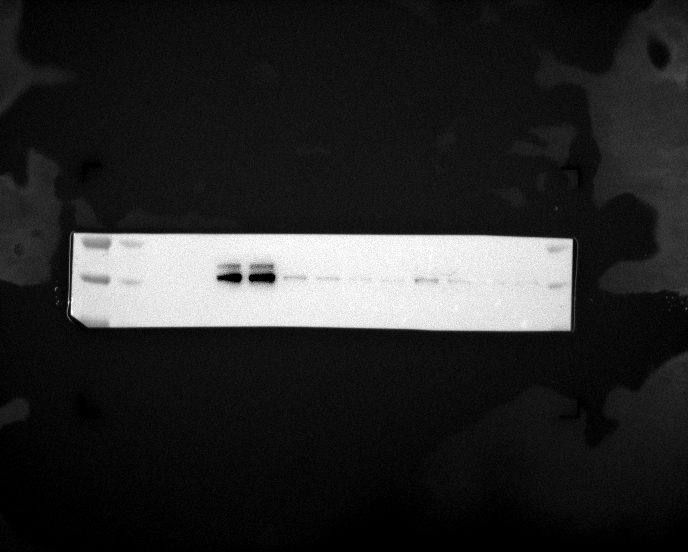


3E-4-ERK


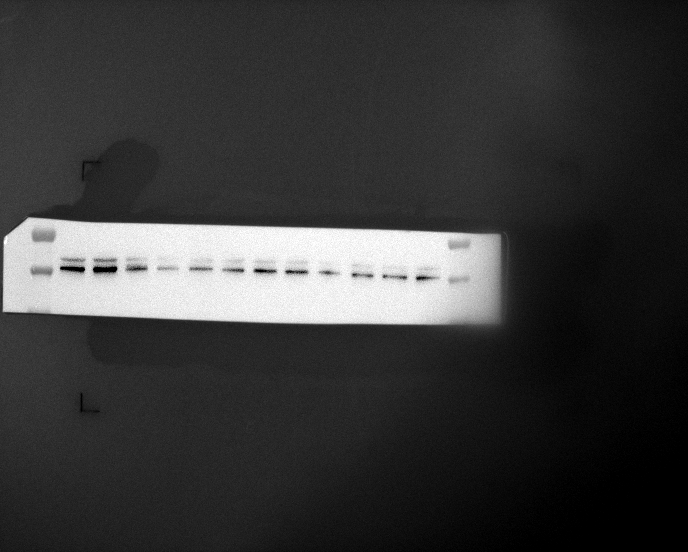


3E-5-PJNK


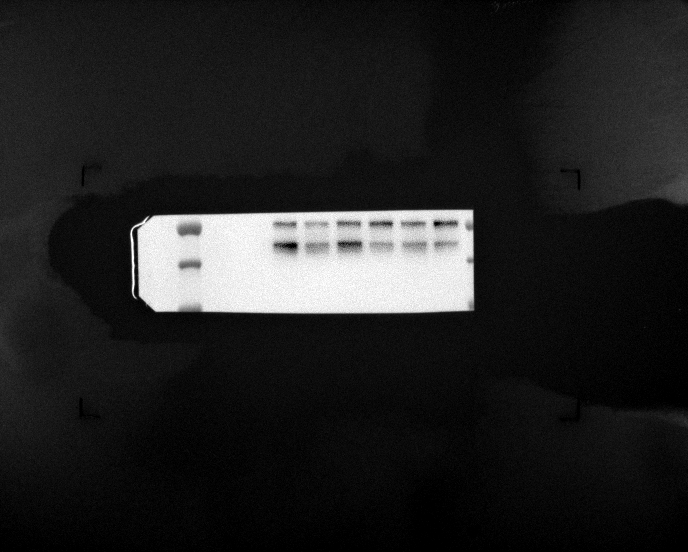


3E-6-JNK


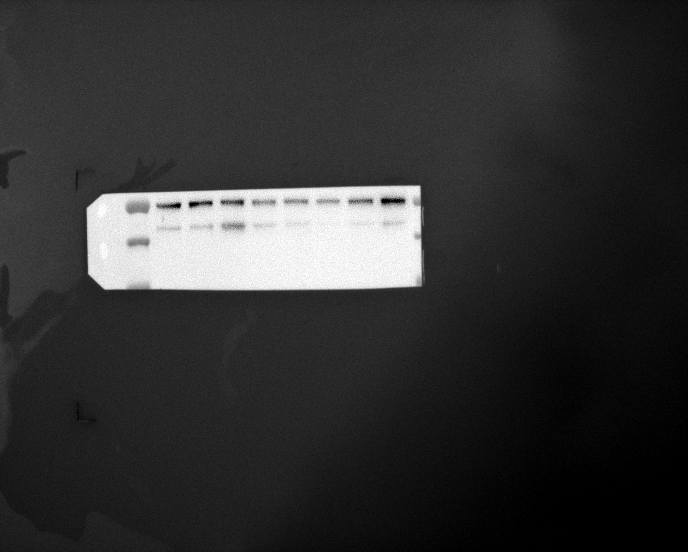


3E-7-PNF-κB


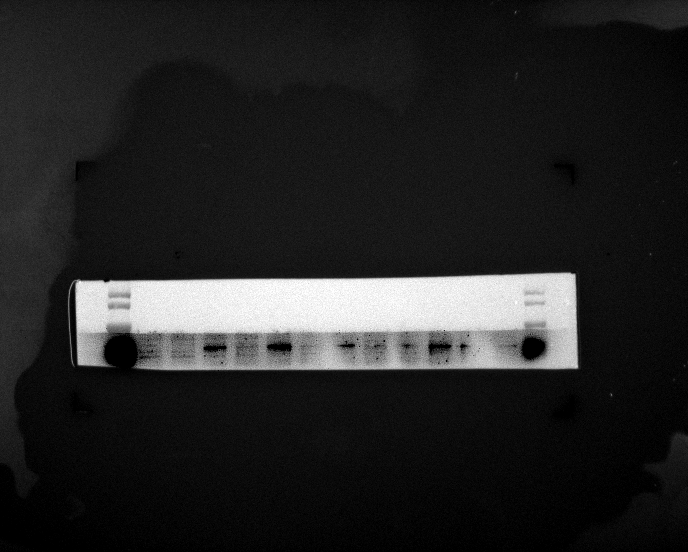


3E-8-NF-κB


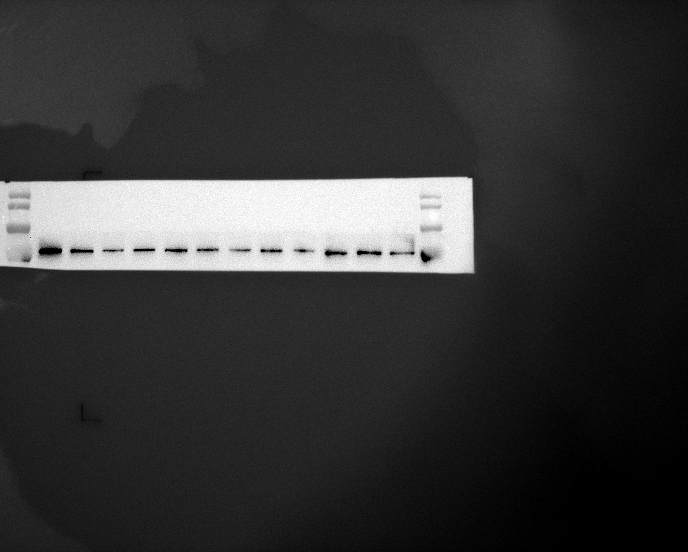


3E-9-GAPDH


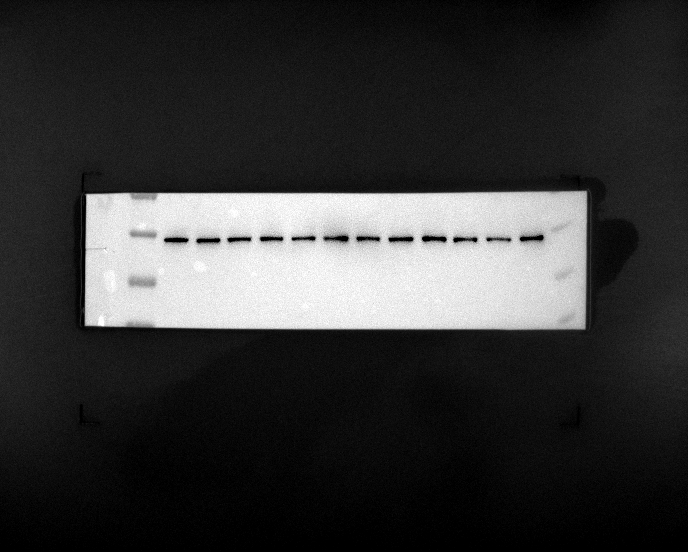


1. ( Figure3F of article)

3F-1-CD147


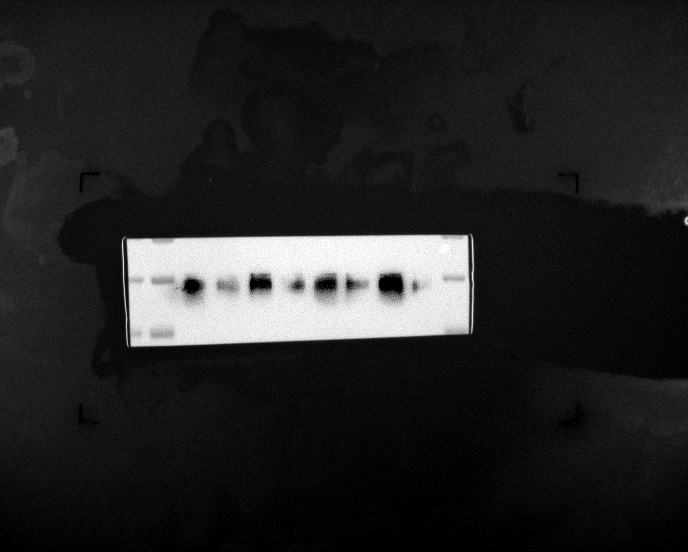


3F-2-Pp38


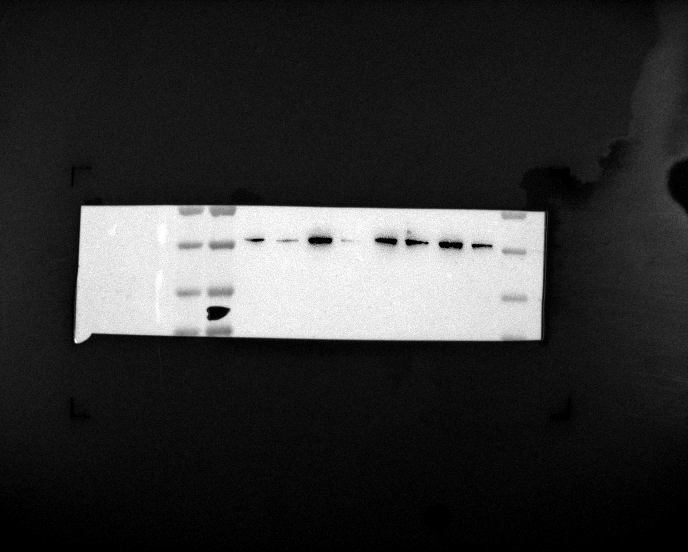


3F-3-p38


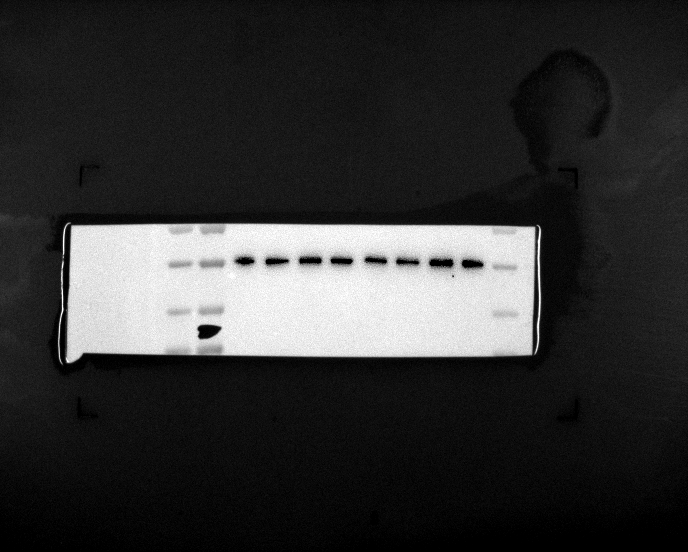


3F-4-PERK


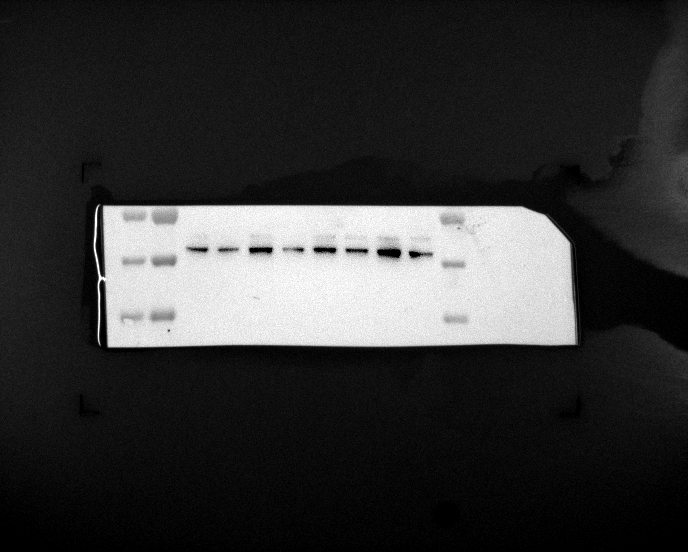


3F-5-ERK


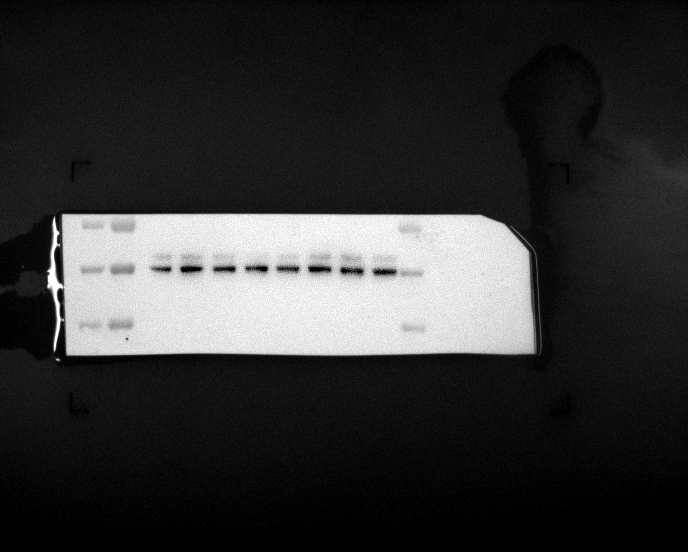


3F-6-PJNK


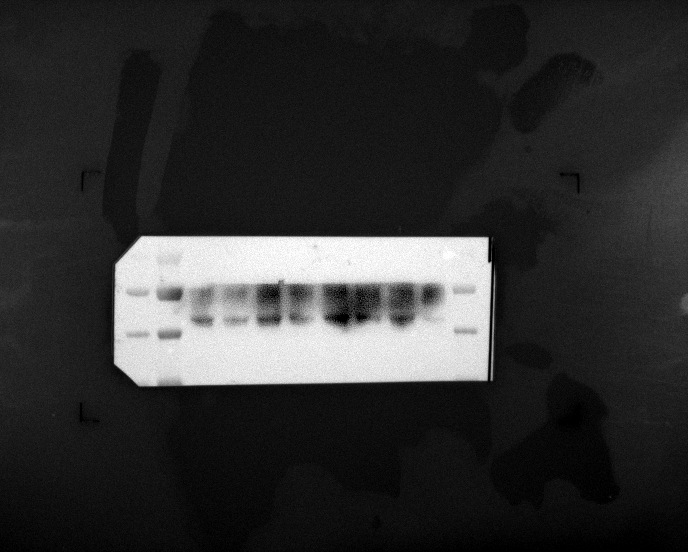


3F-7-JNK


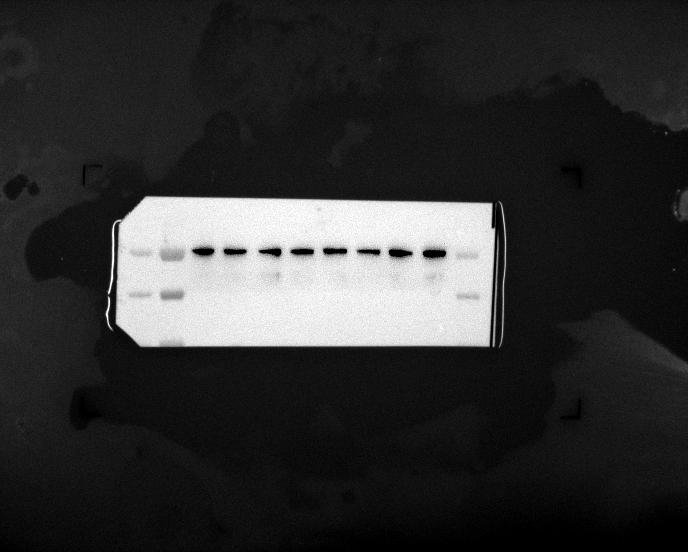


3F-8-PNF-κB


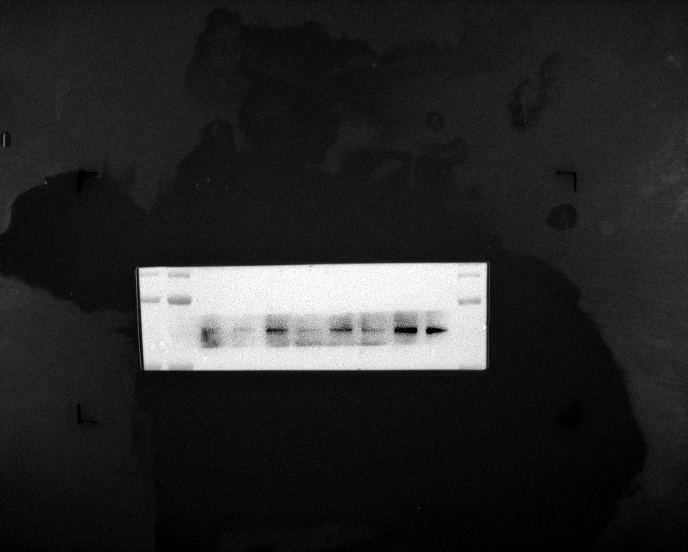


3F-9-NF-κB


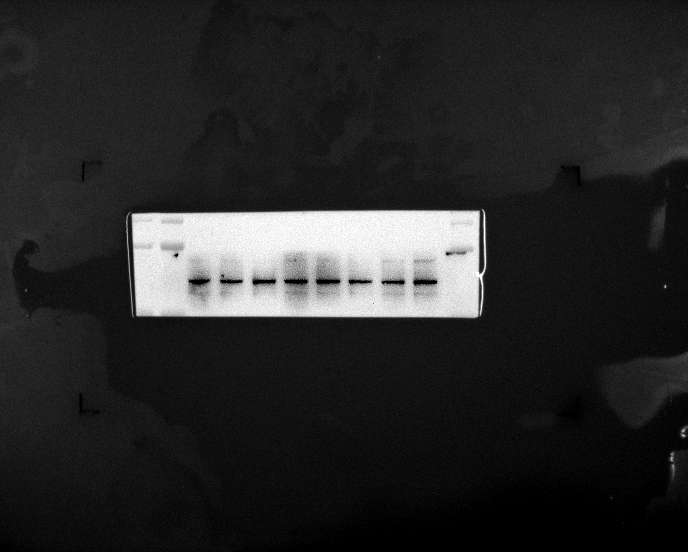


3F-10-GAPDH


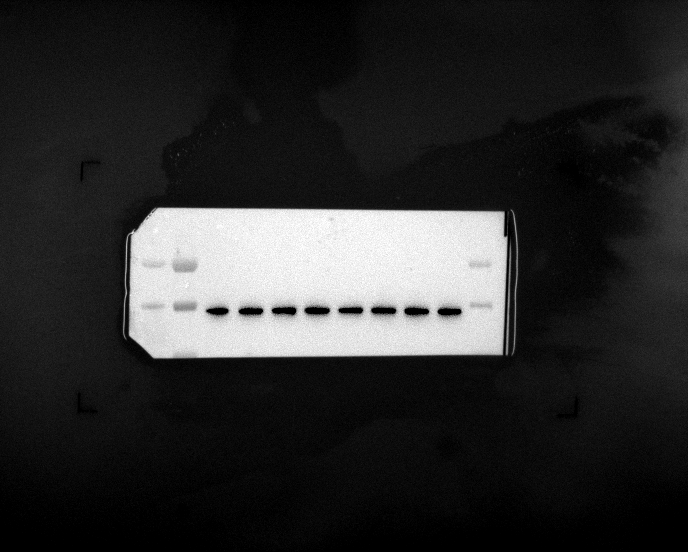

Supplement: Supplementary file 2 [file Table_1.DOC]
